# Gene expression landscape of the *Brassica napus* seed reveals subgenome bias in both space and time

**DOI:** 10.1093/plphys/kiaf283

**Published:** 2025-06-28

**Authors:** Dylan J Ziegler, Deirdre Khan, Jenna L Kalichuk, Michael G Becker, Asher Pasha, Nicholas J Provart, Mark F Belmonte

**Affiliations:** Department of Biological Sciences, University of Manitoba, Winnipeg, MB R3T 2N2, Canada; Department of Biological Sciences, University of Manitoba, Winnipeg, MB R3T 2N2, Canada; Department of Biological Sciences, University of Manitoba, Winnipeg, MB R3T 2N2, Canada; Department of Biological Sciences, University of Manitoba, Winnipeg, MB R3T 2N2, Canada; Department of Cell & Systems Biology/Centre for the Analysis of Genome Evolution and Function, University of Toronto, Toronto, ON M5S 3B2, Canada; Department of Cell & Systems Biology/Centre for the Analysis of Genome Evolution and Function, University of Toronto, Toronto, ON M5S 3B2, Canada; Department of Biological Sciences, University of Manitoba, Winnipeg, MB R3T 2N2, Canada

## Abstract

*Brassica napus* (canola; A^n^A^n^C^n^C^n^) contains both complete diploid genomes from its progenitors *Brassica rapa* (A^n^) and *Brassica oleracea* (C^n^). Despite growing knowledge of the gene expression landscape of the *B. napus* seed, little is known about subgenome bias underpinning the development of specific cells and tissues across the seed lifecycle. Here, we present a large-scale transcriptome atlas of the *B. napus* seed, including both the maternal seed coat and filial embryo and endosperm subregions. We report on extensive, global C^n^ subgenome bias throughout development and use homoeologous gene pairs to describe how subgenomic bias differs across subregions. We find that subgenome bias is most prominent during early development and that the maternal subregions experience far more asymmetric transcript accumulation in favor of the C^n^ subgenome. In particular, the unexpectedly distinct transcriptome profile of the chalazal pole indicates the unique developmental processes involved within the chalaza. Furthermore, we report that genes integral to seed storage comprise a large portion of the transcriptome of mature seeds, especially within the embryo, and that gene pairs previously documented to be instrumental in seed development exhibit low transcriptional bias. This work represents an important synthesis of polyploid transcriptomics in seed biology and provides a comprehensive overview of the *B. napus* gene expression landscape in both space and time.

## Teaser

*Brassica napus* seed development is a complex yet elegant process, marked by dramatic shifts in gene expression across space and time.

## Introduction

Seeds are complex and elegant reproductive structures that provide evolutionary advantages to spermatophytes. In flowers, the seed is generally composed of the embryo that goes on to produce the next sporophytic generation of the plant; the endosperm that supports early embryo growth; and the seed coat that helps to protect the seed (Ziegler et al. 2019). Furthermore, these 3 constituent parts are of different genetic identities. The seed coat originates from the maternal parent while the triploid endosperm and the diploid embryo are products of the unique double fertilization event characteristic of angiosperm seeds (Bleckmann et al. 2014). Seed development must therefore manage these genetic identities to ensure that each constituent part develops simultaneously in tight coordination. This balance has been the subject of many research endeavors in the past, which describe the elongation and development of the seed coat (Radchuk and Borisjuk 2014), the divisions of the embryo (reviewed in Boscá et al. 2011), the cellularization of the endosperm (Batista et al. 2019; Xu et al. 2023), and the cross-talk between the constituent parts (Belmonte et al. 2013; Figueiredo et al. 2015, 2016; Xu et al. 2015; Doll et al. 2020). However, the genetic complexity underpinning seed development is complicated by the extensive polyploid histories of angiosperms. Polyploidy is ubiquitous in land plant evolution, constituting >50% to 70% of all extant flowering plants (Otto and Whitton 2000). While autopolyploidy happens in angiosperms, polyploidy more often occurs as a consequence of interspecific hybridization, which forms complex amphidiploid genomes. *Brassica napus* L. (A^n^A^n^C^n^C^n^) is one such species that formed from the interspecific hybridization of *Brassica rapa* L. (A^r^A^r^) and *Brassica oleracea* L. (C^o^C^o^) approximately 7,500 to 12,500 years ago as a nascent amphidiploid (Chalhoub et al. 2014).

**Figure 1.**
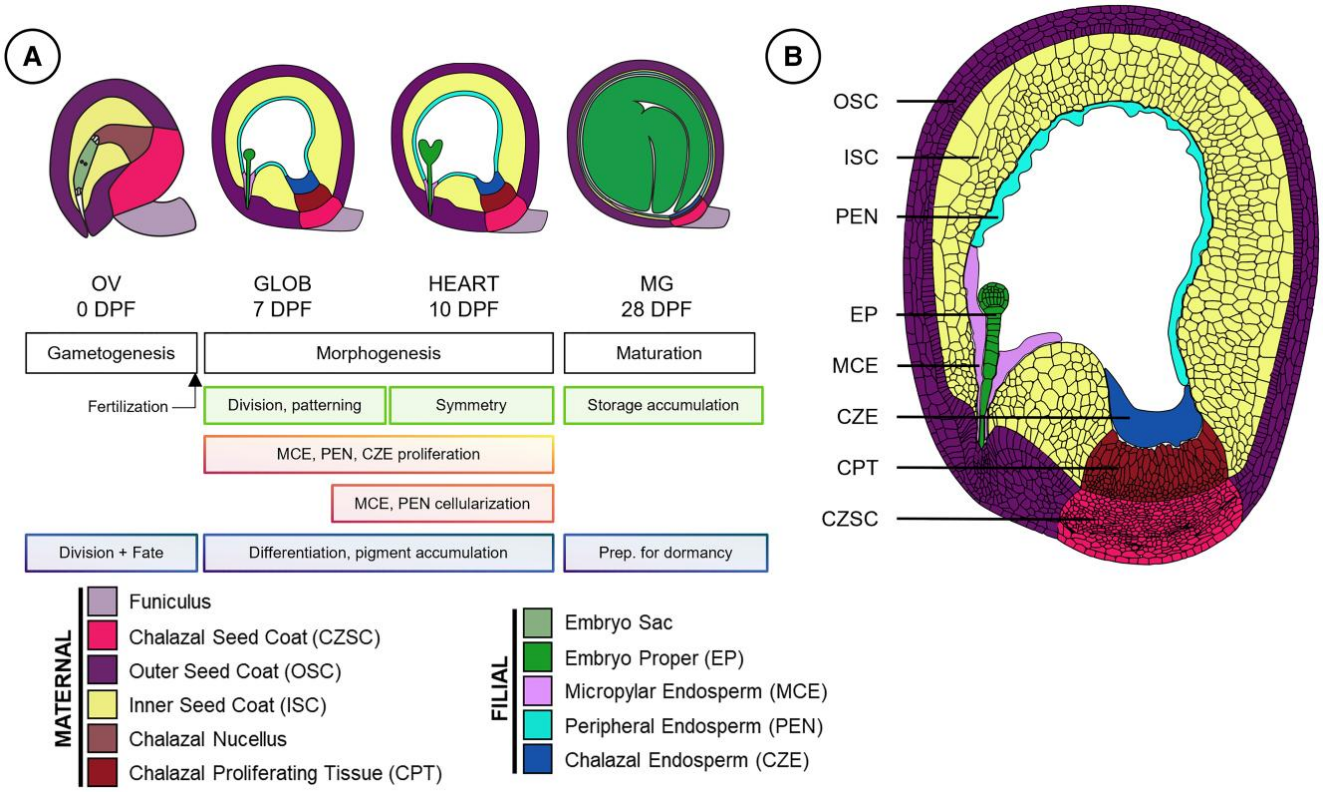
Seed development in *Brassica.*  **A)** Tissue schematic of stages of seed development examined in *B. napus*. Biological processes that occur in different subregions are shown in boxes below. The globular stage seed is expanded **B)**, showing the outer distal seed coat (OSC), inner distal seed coat (ISC), chalazal seed coat (CZSC), chalazal proliferating tissue (CPT), chalazal endosperm (CZE), peripheral endosperm (PEN), micropylar endosperm (MCE), embryo proper (EP), and funiculus.

As one of the world's most important oilseeds, *B. napus* is of great interest to the scientific community. The underlying gene expression landscape required to make a canola seed is poorly understood especially from the earliest stages of seed development. *B. napus* ovules are composed of 2 integuments that encircle the nucellus and female gametophyte. Seed development begins with the ovule (OV) and postfertilization transitions into the globular (GLOB) and then heart (HEART) stages, which constitute the morphogenesis phase (Fig. 1A). Seed storage reservoirs accumulate thereafter, characteristic of mature green (MG) seeds during the maturation phase. As the *B. napus* seed develops, it must coordinate the development of the maternal and filial subregions to orchestrate seed development.

Our previous work showed that transcription factor gene activity was dominated by the A^n^ subgenome in the whole *B. napus* seed (Khan et al. 2022). Furthermore, subgenome bias of nonprotein-coding genome elements like DNA methylation and siRNAs is more frequently biased toward the C^n^ subgenome (Ziegler et al. 2023). Despite the growing body of evidence supporting subgenome bias in *B. napus*, we have yet to explore the possibility of subgenome bias in specific cells and tissues across all of seed development at the mRNA level genome wide. Some recent studies have reported gene expression profiling of *B. napus* seed subregions like the embryo, endosperm, and seed coat (Liao et al. 2019; Ziegler et al. 2019; Gao et al. 2022; Wei et al. 2022). However, these studies do not enable a complete understanding of temporal subgenome bias since none reported on the potential contribution of each subgenome to *B. napus* seed development from fertilization to maturation.

Our dissection of the *B. napus* gene expression landscape and histological characterization of the seed reveals integrated biological processes that underpin seed development. Data revealed each subregion of the seed exhibits distinct transcriptional profiles in the context of subgenome bias. In particular, we found that the chalazal proliferating tissue (CPT) is distinct from the other subregions, likely due in part to its ontogenetic relationship with the nucellus. We also report that specific gene homoeologs involved in seed storage accumulation are expressed asymmetrically between the 2 subgenomes. Analysis of the ancestral subgenomes of this allopolyploid provides seminal evidence into the contributions of each subgenome that define cell and tissue-specific compartments of both maternal and filial regions that contribute to the making of a seed.

## Results

### The *B. napus* seed is a complex yet elegant reproductive structure that can be divided into regions and subregions

We used laser microdissection (LMD) coupled with global RNA sequencing (RNA-seq) to profile the gene expression landscape of the *B. napus* seed. Cells and tissues were captured at the ovule (ov), globular (g, 7 d postpollination [DPP]), heart (h, 10 DPP), and mature green (mg, 28 DPP) stages of development. We profiled gene expression of the maternal seed coat subregions including the inner seed coat (ISC), outer seed coat (OSC), and chalazal seed coat (CZSC), and the CPT. The micropylar endosperm (MCE), peripheral endosperm (PEN), chalazal endosperm (CZE), and the embryo proper (EP) were captured from the filial regions of the seed (Fig. 1A). The EP was further dissected into the embryonic root (ROOT) and cotyledons (COT) in mg seeds. Hereafter, the seed subregion and its corresponding stage are referred to with abbreviations of the stage of development followed by the subregion (i.e. ovCPT refers to the CPT of ovules). Furthermore, while ovules are defined as the female gametophyte encircled by elongating integuments that later fuse to form the seed coat, they are referred to as “seed coat” subregions for consistency in text.

*B. napus* ovules have a distinct chalazal receptacle where the CZSC is composed of smaller condensed cells and the cells of the ISC and OSC are comparatively isodiametric with a palisade layer at the interface of the two. Postfertilization, the elongation of the fusing seed coat migrates the micropylar pole to be parallel to the chalaza with the MCE encircling the suspensor at the base of the EP (Fig. 1B). The endosperm forms a thin syncytium surrounding the inner periphery of the ISC and is referred to as the PEN. The endosperm additionally forms a syncytial cyst at the chalazal pole, referred to as the CZE. At the interface between the CZE and CZSC lies the maternally derived CPT. The CPT in *B. napus* proliferates to a large mass of cells at the interstice of the filial CZE and the maternal CZSC postfertilization (Fig. 1B).

### The C^n^ subgenome asymmetrically accumulates transcripts across seed development

**Figure 2.**
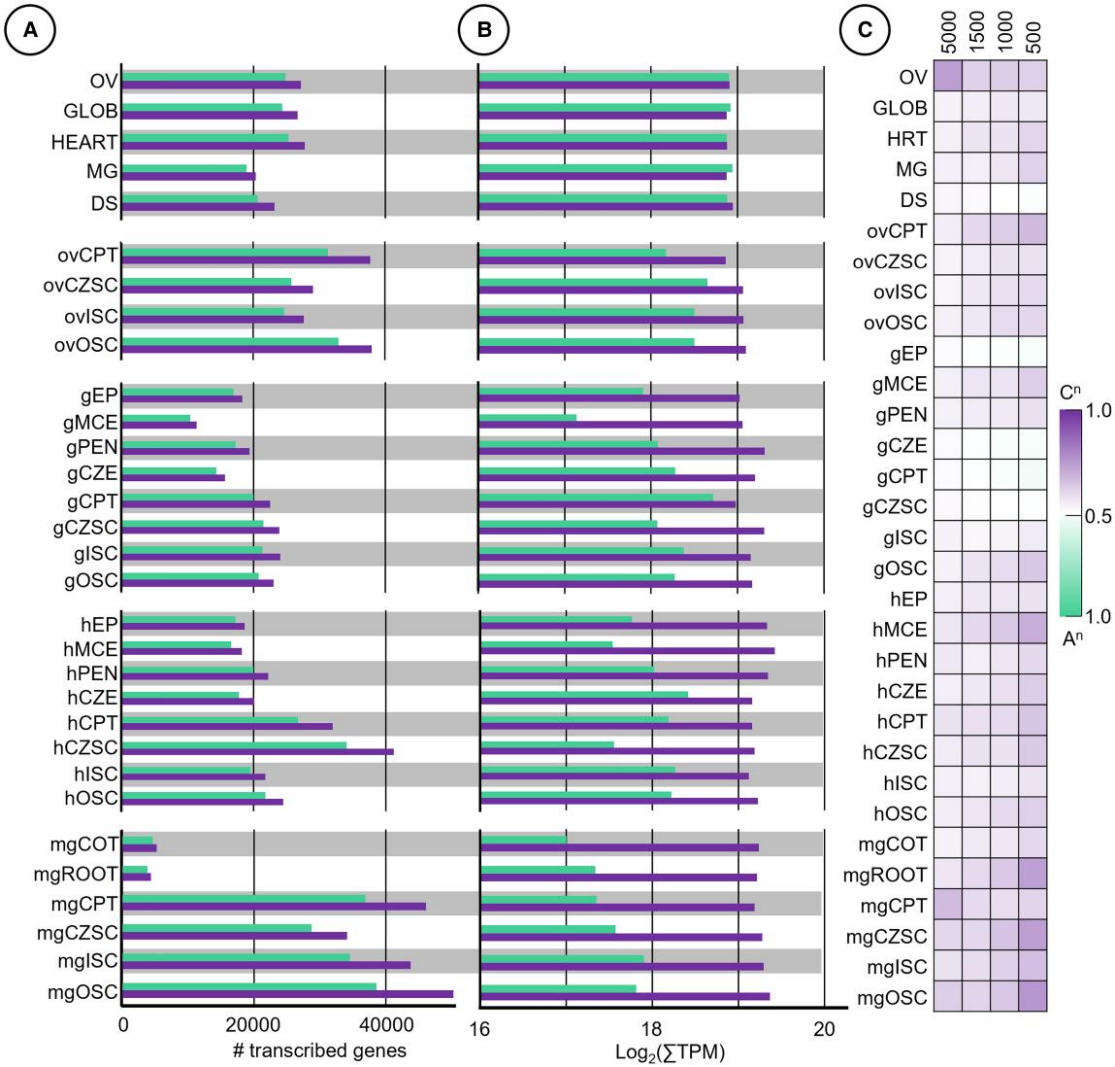
Transcript accumulation of subgenome across space and time. Subgenome representation spatially and temporally as **A)** number of transcribed genes (TPM > 1), **B)** summed TPM (middle), and **C)** proportion of genes expressed in the top 5,000, 1,500, 1,000, and 500 genes belonging to each respective subgenome (right).

Collectively, we identified 55,839 genes in the LMD data set that were not detected in the whole seed thus increasing transcript detection by 44% (Supplementary Fig. S1). This total accounts for 78.6% of all annotated genes in the *B. napus* cv. Topas reference genome (Chalhoub et al. 2014). Marker genes that are well supported in the literature to be subregion specific like *TRANSPARENT TESTA16* (seed coat), *LEAFY COTYLEDON1* (endosperm, globular EP), *ZHOUPI* (endosperm) (Nesi et al. 2002; Yang et al. 2008; Song et al. 2021), and genes shown to be subregion specific in Arabidopsis (*MIRO3* And *KINESIN-LIKE 14* [*KIN14T*]; Belmonte et al. 2013) all show compartment specificity in our own data set (Supplementary Fig. S2). Genes belonging to the C^n^ subgenome were overrepresented compared to the A^n^ subgenome at both the level of detected transcribed genes (Fig. 2A) and the total sum of transcript activity as represented by transcripts per million (TPM) (Fig. 2B). The seed subregions were universally biased toward the C^n^ subgenome compared to the whole seed, with both more genes detected and higher transcript accumulation from the C^n^ subgenome. At the genome level, subgenome bias of total TPM ranged from 48.9% A^n^/51.1% C^n^ in the DS to 51.1% A^n^/48.9% C^n^ in MG seeds. In contrast, subgenome bias within subregions was most asymmetric in the mgCOT (17.6% A^n^/82.4% C^n^) and most equivalent in the gCPT (45.4% A^n^/54.5% C^n^) (Fig. 2B). Furthermore, the 500, 1,000, 1,500, and 5,000 most highly transcribed genes across all stages of seed development as well as in all subregions were either not biased toward any subgenome or biased to the C^n^ subgenome (Fig. 2C). At the broad transcriptomic level, C^n^ subgenome bias was ubiquitous in all seed subregions, both in genes detected and in total transcripts.

### Subgenome bias of homoeologous gene pairs was most divergent in the ovCPT compared to all other maternal subregions

**Figure 3.**
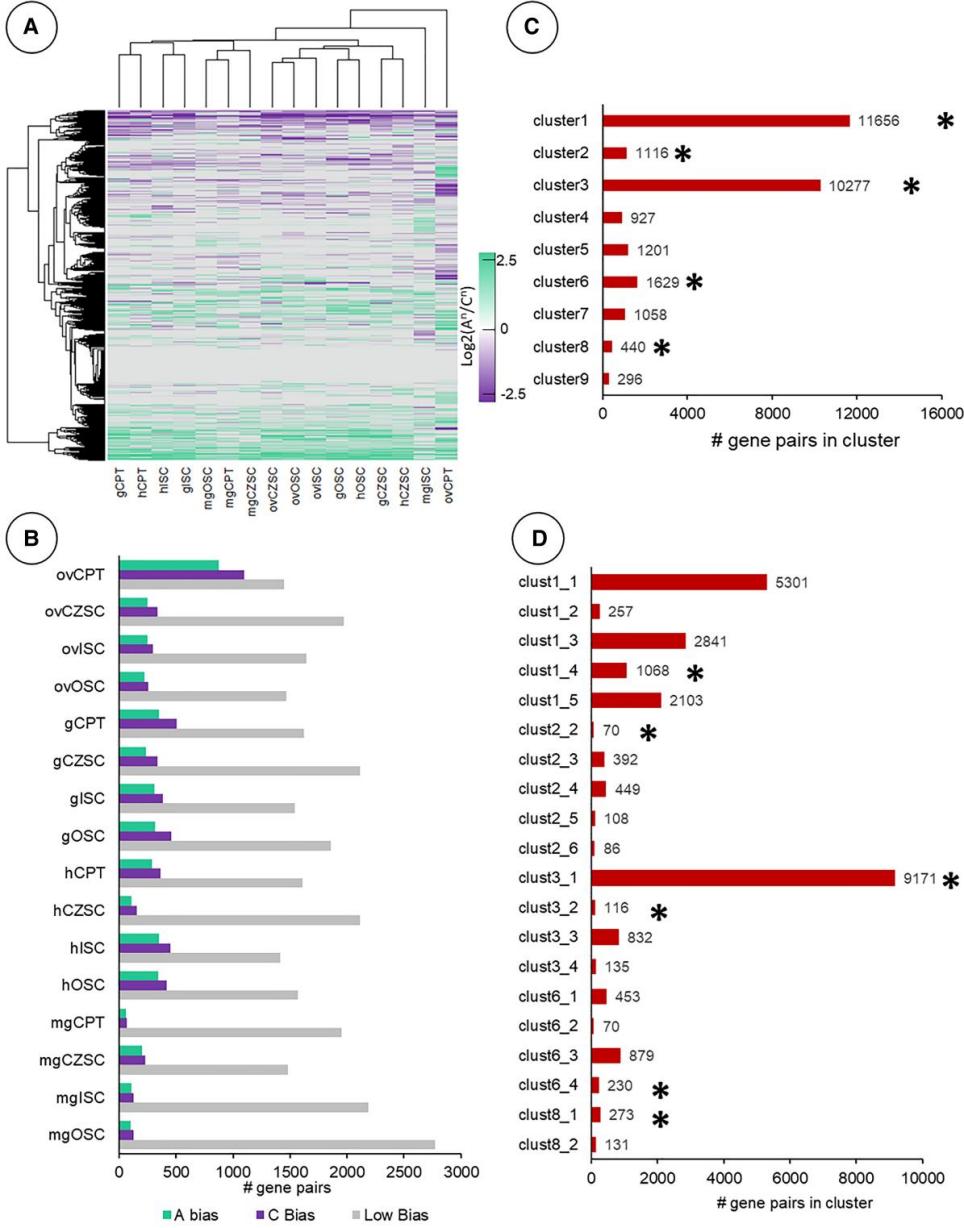
Subgenome bias and clustering analysis of maternal subregions in the *B. napus* seed. **A)** Subgenome bias of gene homoeologues expressed as Log_2_(A^n^/C^n^) of their respective TPM values. Euclidean distance was used to compute distance between data points. **B)** Number of gene pairs exhibiting strong genomic bias (Log_2_(A^n^/C^n^) ≥ 2 [“A Bias”] or Log_2_(A^n^/C^n^) ≤ −2 [“C Bias”]) or “Low Bias” (0.1 ≥ Log_2_(A^n^/C^n^) ≥ −0.1). **C)** Gene pair list from **A)** clustered into *k* = 9 groups, number of gene pairs are tabulated, and heat maps of all clusters available as Supplementary Fig. S2. Gene pair clusters indicated by an asterisk (*) are carried forward into further analysis in **D)**. Clusters 1, 2, 3, 6, and 8 were then clustered again to *k* = 5, *k* = 6, *k* = 5, *k* = 4, and *k* = 3 clusters, respectively, to parse the gene lists. Clusters in **D)** with <60 gene pairs were removed from the bar plot but included within Supplementary Data Set S1. Clusters in **D)** indicated with an asterisk (*) were used in the GO analysis of Fig. 4.

To further assess subgenome bias in the maternal subregions, we clustered homoeologous gene pairs between the A^n^/C^n^ subgenomes based on their expression levels relative to each other (Fig. 3A; Supplementary Fig. S3 and Data Set S1). The ovCPT was the most distinct subregion, clustering away from all other stages and subregions. The CPT in seeds undergoing morphogenesis clustered together and shared a larger clade with the ISC and OSC subregions. The mgCPT, in contrast, clustered alongside the mgOSC and the mgCZSC. The ovCPT additionally had the most gene homoeologs with high subgenome bias toward the C subgenome (Log_2_(A^n^/C^n^) ≥ 2 or Log_2_(A^n^/C^n^) ≤ −2) compared to all other seed subregions and developmental stages (Fig. 3B).

**Figure 4.**
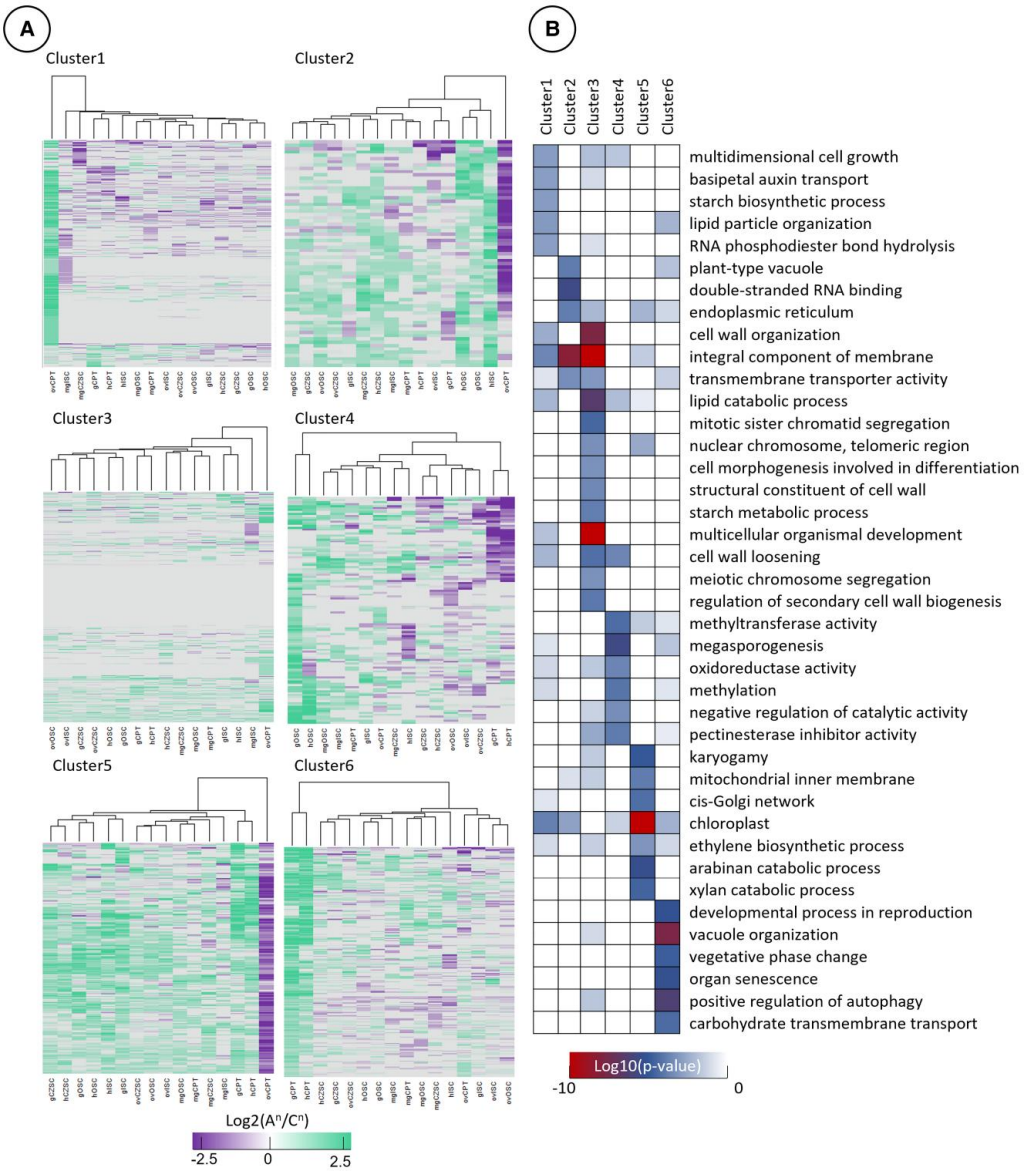
GO analysis of gene clusters in maternal subregions. **A)** Clusters of the data in Fig. 3C, selected for subgenome bias in certain stages or lack thereof. **B)** GO enrichment of the clusters selected in **A)**, wherein statistically significant GO terms were only selected if >1 homologous gene pair constituted the GO term. Complete GO analysis is documented within Supplementary Data Set S2.

Next, we broke down this cluster analysis to examine gene sets with patterns of subgenome bias that may contribute to the conspicuous asymmetry between the 2 subgenomes (Fig. 3, C and D). We selected these clusters for downstream analysis based on (i) extensive bias in 1 or 2 subregions at certain developmental stages (Fig. 4A, Clusters 1 and 6); (ii) gene lists with homoeologous pairs in which subregions were visually distinct from the other maternal subregions (Fig. 4A, Clusters 2, 4, and 5); or (iii) clusters with minimal subgenome bias (Fig. 4A, Cluster 3). We then performed Gene Ontology (GO) enrichment analysis on these gene clusters to investigate the biological processes influenced by subgenome bias (Supplementary Data Set S2).

### Maternal subregions in early seed development are more transcriptomically divergent than that of mature seeds

GO terms associated with cell growth and hormone transport were significantly enriched (*P* < 0.001) in the cluster with ovCPT A^n^ subgenome bias (Fig. 4B, Cluster 1). Clusters with ovCPT C^n^ subgenome bias had enriched GO terms for endomembrane systems and the vacuole in addition to polysaccharide catabolism (arabinan and xylan), karyogamy, and mitochondrial/chloroplast (Fig. 4B, Clusters 2 and 5). Clusters 4 and 6, which segregated out gCPT and hCPT together as their own clade with subgenome bias distinct from other subregions, were significantly enriched in GO terms relating to epigenetic maintenance, pectin biochemical processing, vacuole organization, autophagy, and organ senescence (Fig. 4B). These clusters were also enriched for biological processes involved in reproduction and reproductive transitions and phase changes. Conversely, the cluster characterized by low subgenome bias was most significantly enriched for processes associated with cell wall organization and loosening, secondary cell wall biogenesis, and genes relevant to mitotic/meiotic processes (Fig. 4B, Cluster 3). At the whole transcriptome level, the CPT was the most different subregion and had the most divergent pattern of subgenome bias compared to the other subregions at any stage of development.

### The CPT is derived from the nucellus and is transcriptomically distinct from other maternal subregions of the seed

**Figure 5.**
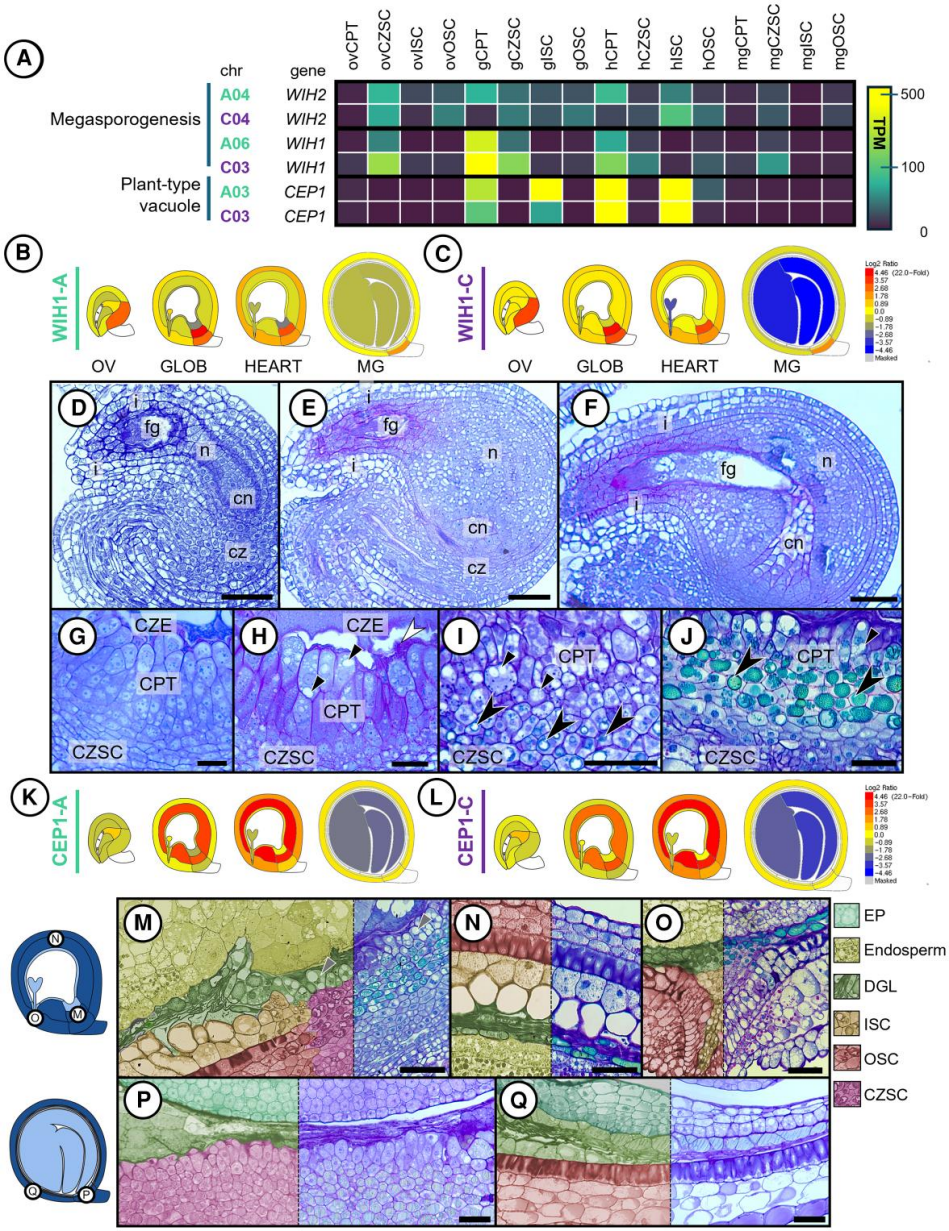
Developmental shifts and cell elimination within the CPT and ISC through seed development. **A)** Heat map of selected homologous gene pairs belonging to significantly enriched GO terms from the clusters in Fig. 4 as they pertain to the developmental shifts within the CPT. **B)** Relative expression visuals from ePLANT expressed as Log_2_(TPM of *WIH1*/(median TPM of *WIH1* across all subregions) of *WIH* genes from both A^n^ and **C)** C^n^ subgenome homoeologues spanning OV, GLOB, HEART, and MG seeds. **D)** Developmental series (stained with toluidine blue and periodic acid and Schiff's reagent) of the early ovule enveloped by both elongating integuments (i) with the tenuinucellate nucellus (*n*) surrounding the female gametophyte (fg). The nucellus extends from the basal chalazal nucellar body (cn), adjacent to the chalaza (cz). **E)** maturing ovule, **F)** ovule with the cellularized mature fg subtended by the cn, and **G)** 7 DPP seed chalaza divided into the CPT, CZE, and CZSC. **H)** 10 DPP seed chalaza with the nucellar lysate (white arrowhead) at the interstice of the CZE and CPT with fragmented vacuoles (black arrow) now populating the cells of the CPT. **I)** Chalaza at 14 DPP with the incipient deposition of polyphenols in the pigment layer (black arrowheads) constituted of the CZSC and the proximal cell layers of the CPT. **J)** 28 DPP seed chalaza. **K)** Relative expression visuals from ePLANT expressed as Log_2_(TPM of *CEP*/(median TPM of *CEP1* across all subregions) of *CEP1* genes from both A^n^ and **L)** C^n^ subgenome homoeologues spanning OV, GLOB, HEART, and MG seeds. **M)** Partially false-colored sections illustrated the degenerating cell layer (DGL) of seeds 14 DPP at the **M)** chalaza, **N)** peripheral seed coat, and **O)** micropylar pole. **P)** 21 DPP seeds at the **P)** chalazal pole and **Q)** peripheral seed coat. Bar = **D)** 50 *µ*m, **E)** 50 *µ*m, **F)** 75 *µ*m, **G)** 50 *µ*m, **H)** 50 *µ*m, **I)** 30 *µ*m, **J)** 25 *µ*m, **M)** 50 *µ*m, **N)** 50 *µ*m, **O)** 60 *µ*m, **P)** 50 *µ*m, and **Q)** 55 *µ*m.

Clustering of homoeologous gene activity implied that the CPT is a distinct subregion of the seed. We examined the CPT gene lists used for GO enrichment in Fig. 4 and mined the data for genes relevant to seed development and reproduction. Two GO terms relating to megasporogenesis and vacuole modification identified statistically significant gene homoeologs associated with reproductive development. Among them, a cysteine endopeptidase (*CEP1*) was largely expressed only within the ISC and CPT during morphogenesis (Fig. 5A). The *WINDHOSE* (*WIH*) gene homoeologs were largely transcribed within the CZSC in ovules, followed by a dramatic increase in transcript accumulation of *WIH1* in the ovCPT, and finally with declining transcript abundance of both homoeologous gene pairs in the CPT and CZSC of HEART and MG stage seeds (Fig. 5, B and C). We developed an eFP Bio-Analytic Resource for *B. napus* to visualize gene expression across seed development in every seed subregion.

We examined the anatomical development of the CPT at the early ovule stage through to seed maturation. In early ovules, the nucellus proliferated as a single cell layer encircling the developing female gametophyte (Fig. 5D). The nucellar body proliferated shortly afterward and the chalazal nucellus localized adjacent to the developing CZSC (Fig. 5E). The chalazal nucellus then elongated and contacted at the antipodal end of the mature female gametophyte (Fig. 5F). At the globular stage, the cells of the chalazal nucellus form conspicuous nuclei, elongate, and increase cytoplasmic density (Fig. 5G). Seeds at the heart stage accumulated fragmented vacuoles (Fig. 5H). A distinct cellular lysate of the remnant CPT cells was seen at the interface between the CPT and the CZE in both GLOB and HEART seeds (Fig. 5F). This lysate accumulated as more cell layers of the CPT were lost to the expanding seed. By 14 DPP, the remaining CPT cells were less distinct from the CZSC (Fig. 5I). The vacuolar fragments accumulated pigment in both the CPT and CZSC once the seed had initiated maturation. The cell layers of the CPT proximal to the CZSC accumulated pigment by 14 DPP, and the remaining layers of the CPT were entirely subsumed into the pigment layer by 28 DPP (Fig. 5J). *CEP1* gene activity is high in the inner maternal regions of the seed during morphogenesis (Fig. 5, K and L). The pigment layer matures from the innermost cell layers of the ISC and forms a peripheral boundary to the OSC by 14 DPP (Fig. 5, M to O). The CPT and CSZC were incorporated into this layer immediately prior to the initiation of maturation, at 21 DPP (Fig. 5, P and Q). Cells of the CPT and ISC not incorporated into the mature pigment layer collapsed and formed a degenerating layer. *CEP1* gene activity is significantly enriched (*P* < 0.001) in ISC and CPT subregions in morphogenesis and readily declines in MG seeds. These 2 subregions lose the majority of their viable cells by 28 DPP, with the rest of the cells being largely incorporated into the pigment layer or crushed into a cellular lysate. The CPT, therefore, begins seed development with a unique subgenome bias profile from the rest of the maternal subregions and proceeds through seed development altering its anatomy and transcriptome to facilitate dynamic functionality as the seed matures.

### The ISC and OSC are genetically and anatomically distinct undergoing different developmental milestones

**Figure 6.**
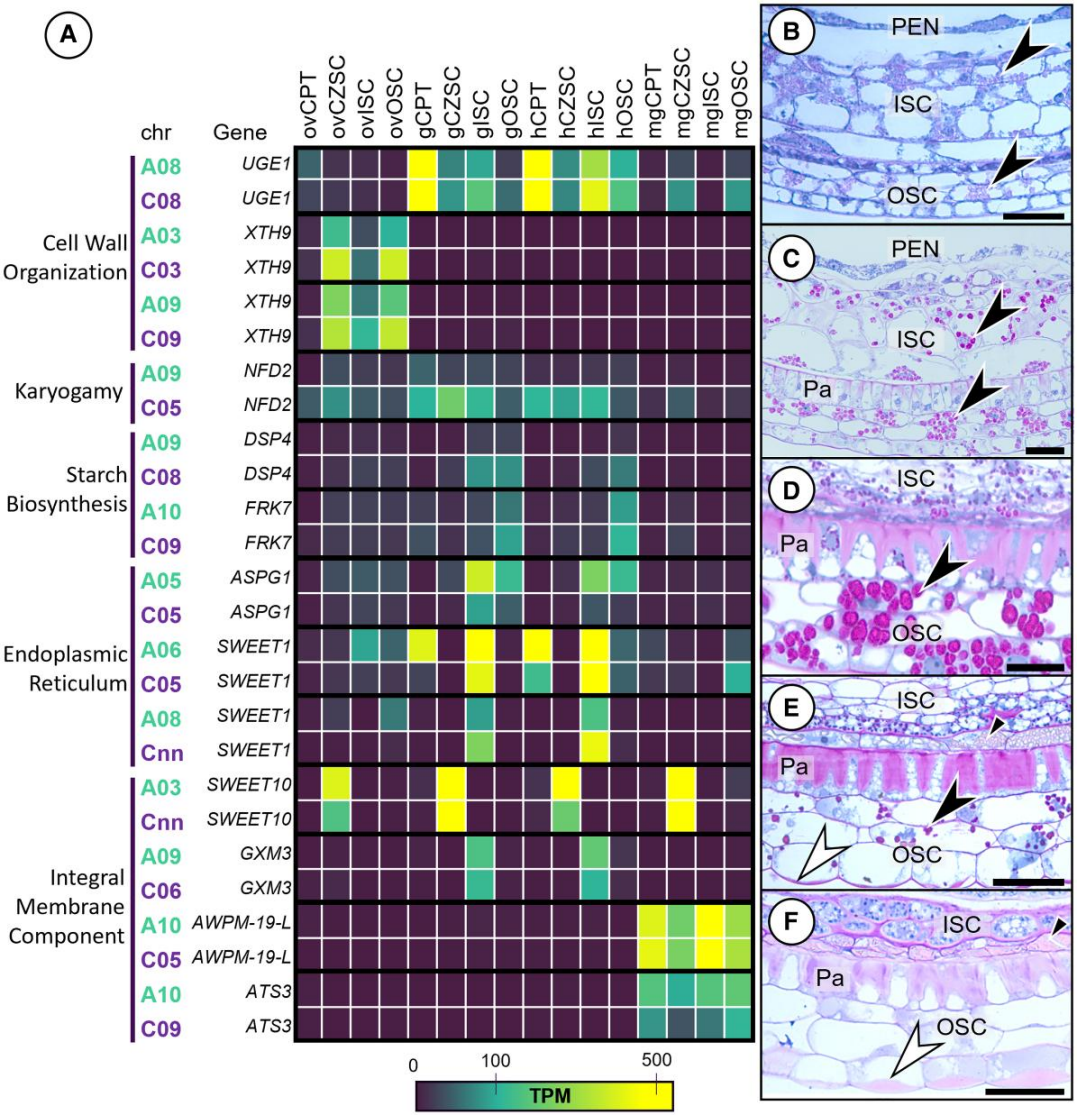
Transcription of genes associated with polysaccharide metabolism and cell wall development in the seed coat. **A)** Heat map of selected homologous gene pairs belonging to significantly enriched GO terms from the clusters in Fig. 4 as they pertain to seed coat development. Longitudinal sections of the developing seed coat (stained with amido black and periodic acid and Schiff's reagent) show the progressive accumulation of starch granules (black arrowheads) in the ISC and OSC by 7 DPP **B)**, 10 DPP **C)**, and 14 DPP **D)**. Starch reserves dissipate at **E)** 21 DPP, and the ISC accumulates the pigment layer (black arrows) directly beneath the palisade layer of the OSC. Starch reserves are entirely absent by 28 DPP **F)**. The anticlinal walls of the palisade (Pa) layer of the OSC gradually thicken as the seed develops, and the primary walls of the ISC (black arrowheads) also expand by 28 DPP. Mucilage (white arrowheads) accumulates in the outer periclinal walls of the OSC epidermis. Bar = **B)** 50 *µ*m, **C)** 40 *µ*m, **D)** 40 *µ*m, **E)** 50 *µ*m, and **F)** 50 *µ*m.

Many genes responsible for cell wall remodeling, cell elongation, and deposition of storage materials were transcriptomically isolated to specific developmental stages and within specific subregions in the seed. Among them, genes implicated in cell wall organization accumulated in the ovule seed coat (*XTH9* endoxyloglucan transferase) and in the seed coat during morphogenesis (*UGE1,* UDP-galactose epimerase) (Fig. 6A). *XTH9* transcripts were more abundant in the C^n^ subgenome in all 3 ovule seed coat subregions and across 2 sets of gene homoeologs. *UGE1* transcript accumulation was biased toward the C^n^ subgenome in the gISC, hOSC, and mgCZSC/OSC. C^n^ bias persisted in transcripts associated with nuclear fusion (*NUCLEAR FUSION DEFECTIVE 2* [*NFD2*]), starch biosynthesis (*PHOSPHOGLUCAN PHOSPHATASE* [*DSP4*] and *FRUCTOKINASE 7* [*FRK7*]), and the bidirectional monosaccharide transporter (*SWEET1*) in the mgOSC. A^n^ subgenome bias was apparent in the homoeolog pair of *ASPARTIC PROTEASE OF GUARD CELL 1* (*ASPG1*) in the gISC/OSC and hISC/OSC, *SWEET10* in the ovCZSC and hCZSC, and in the *ATS3* pair (*EMBRYO-SPECIFIC PROTEIN 3*) at seed maturation.

Histological analysis of the seed coat revealed cell wall modifications and polysaccharide accumulation throughout seed development. At 7 DPP, starch granule accumulation was visible throughout the ISC and within the nondermal cells of the OSC (Fig. 6B). Starch deposition increased by 10 and 14 DPP while simultaneously the palisade layer of the OSC underwent robust primary wall thickening leaving the outer periclinal wall largely unmodified (Fig. 6, C and D). Cells of the outermost ISC accumulated pigment within the vacuole by 21 DPP and the starch granules had been nearly entirely utilized by the developing seed in both the ISC and OSC (Fig. 6E). By 28 DPP, cells of the ISC contained protein-rich dense cytoplasm with fragmented vacuoles and were surrounded by conspicuously reinforced primary walls, reminiscent of the late stage CPT (Fig. 6F). The starch reservoirs in both the ISC and OSC were entirely absent at 28 DPP, and mucilage deposits had grown further in the epidermis of the OSC. The ISC and OSC both serve as important nutritive reservoirs for the seed but are developmentally distinct. The ISC undergoes cell wall remodeling and cytosolic protein accumulation during its integration into the pigment layer at the time of maturation. The OSC instead exhausts its reservoirs at the time of maturation and prepares the seed for quiescence by strengthening the primary walls of the palisade layer and deposits hygroscopic mucilage stores within the epidermis to ready the seed for germination. These developmental shifts, especially in regard to starch metabolism and cell wall reconstruction, are controlled in part by genes exhibiting transcriptional bias from 1 subegnome.

### Lipid processing exhibits low subgenomic bias in the maternal subregions of the *B. napus* seed

**Figure 7.**
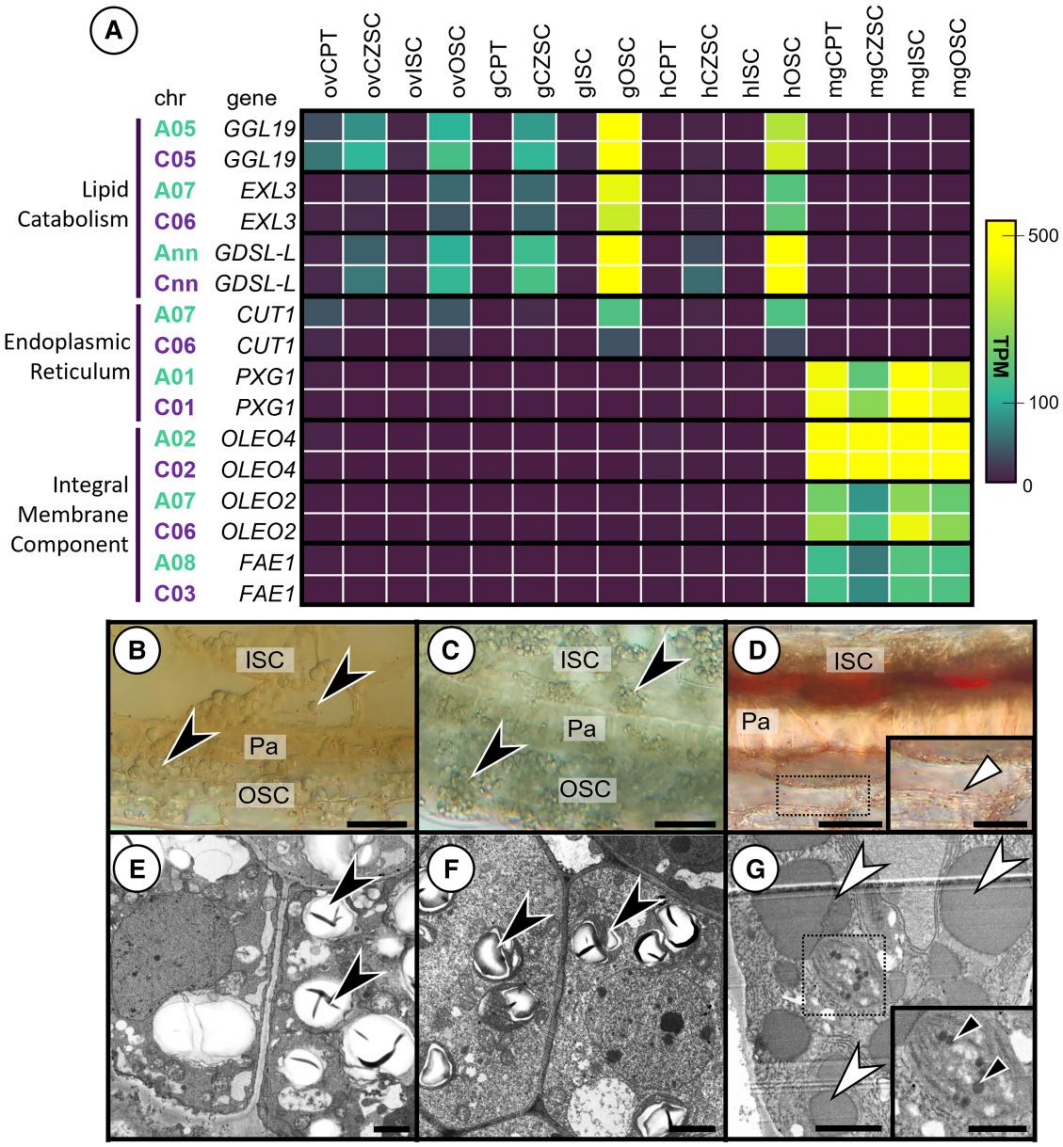
Lipid metabolism and endomembrane associated genes in maternal subregions. **A)** Heat map of selected homoeologues belonging to significantly enriched GO terms from the clusters in Fig. 4 as they pertain to lipid biosynthesis and seed maturation. **B** to **D)** Longitudinal hand sections of the developing seed coat do not stain with Sudan IV at **B)** 7 DPP and **C)** 10 DPP. Starch granules (black arrowheads) instead constitute the carbon source during morphogenesis. **D)** At 28 DPP, lipid accumulation appears in both the ISC and OSC but is absent in the Palisade (Pa) layer. **E** to **G)** TEM micrographs further confirm the presence of starch granules (black arrowheads) throughout the CZSC. Numerous starch granules fill the CZSC cells of both **E)** 7 DPP and **F)** 10 DPP seeds but are eventually used up by **G)** 28 DPP. Fragmented vacuoles (white arrowheads) gradually fill with proanthocyanidins at 28 DPP. Lipid accumulation occurs in plastids (black arrows, **G_inset_**). Bar = **B)** 30 *µ*m, **C)** 30 *µ*m, **D)** 40 *µ*m **D_inset_)** 40 *µ*m, **E)** 2 *µ*m, **F)** 2 *µ*m, **G)**, 2 *µ*m, and **G_inset_)** 1 *µ*m.

While maternal subregions do not accumulate cytoplasmic lipid bodies, lipid homeostasis is important for seed coat waterproofing and maturation. We uncovered gene homoeologs implicated in lipid homeostasis that showed minimal subgenomic bias (Fig. 7A). For example, *CUT1*, a gene implicated in very long chain fatty acid synthesis in dermal tissue, was biased toward the A^n^ subgenome in the gOSC and hOSC. Genes like *EXORDIUM-LIKE 3* (*EXL3*), involved in fatty acid elongation, and the *GUARD CELL-ENRICHED GDSL LIPASE* (GGL) implicated in lipid homeostasis both lack subgenome bias. Furthermore, *FATTY ACID ELONGATION 1* (*FAE1*) was transcribed solely in the MG seed in all subregions. Expectedly, we found genes in the oleosin family to also be highly transcribed in all MG seed subregions with minimal subgenome bias across *OLEO2* and *OLEO4* homoeologs.

During morphogenesis, the seed coat did not accumulate lipid stores at the level detectable with Sudan IV staining (Fig. 7, B and C). Instead, lipids accumulated in the palisade layer of the OSC and the outermost layer of the ISC, the pigment layer, at 28 DPP (Fig. 7D). Starch reservoirs populate the CZSC during seed morphogenesis but are eventually consumed and replaced with vacuole fragments filled with proanthocyanidins (Fig. 7, E to G). Lipids appeared late in seed development in all seed coat subregions—the OSC and CZSC with elaioplasts and the ISC with reinforced suberized walls. As both *OLEO4* and *FAE1* have low subgenome bias in maternal tissues, it seems lipid deposition in seeds does not exhibit substantial subgenome bias. Lipids accumulate at the onset of maturation in multiple forms like waxes or elaioplasts in the maternal subregions, but these processes seem unlikely to be dictated by 1 genome predominantly over another.

### Filial subregions are transcriptomically less distinct in subgenome bias than the maternal subregions

**Figure 8.**
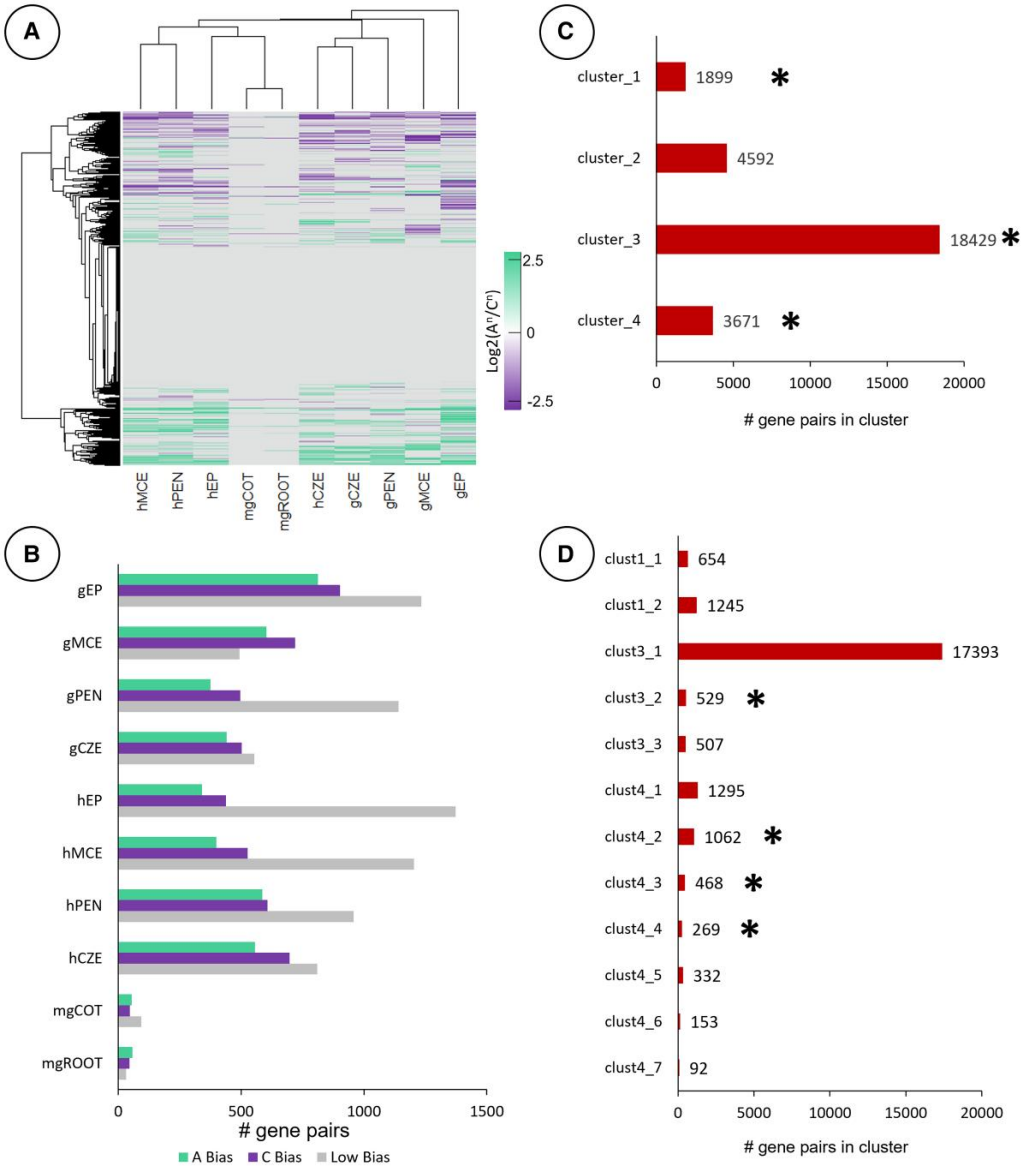
Subgenome bias and clustering analysis of filial subregions in the *B. napus* seed. **A)** Subgenome bias of gene homoeologues expressed as Log_2_(A^n^/C^n^) of their respective TPM values. Euclidean distance was used to compute distance between data points. **B)** Number of gene pairs exhibiting strong genomic bias (Log_2_(A^n^/C^n^) ≥ 2 [“A Bias”] or Log_2_(A^n^/C^n^) ≤ −2 [“C Bias”]) or “Low Bias” (0.1 ≥ Log_2_(A^n^/C^n^) ≥ −0.1). **C)** Gene pair list from **A)** clustered into *k* = 4 groups, number of gene pairs are tabulated, and heat maps of all clusters available as Supplementary Fig. S3. Gene pair clusters indicated by an asterisk (*) are carried forward into further analysis in **D)**. Clusters 1, 2, and 4 were then clustered again to *k* = 2, *k* = 3, and *k* = 7 clusters, respectively, to parse the gene lists. Clusters in **D)** with <60 gene pairs were removed from the bar plot but included within Supplementary Data Set S3. Clusters in **D)** indicated with an asterisk (*) were used in the GO analysis of Fig. 9.

**Figure 9.**
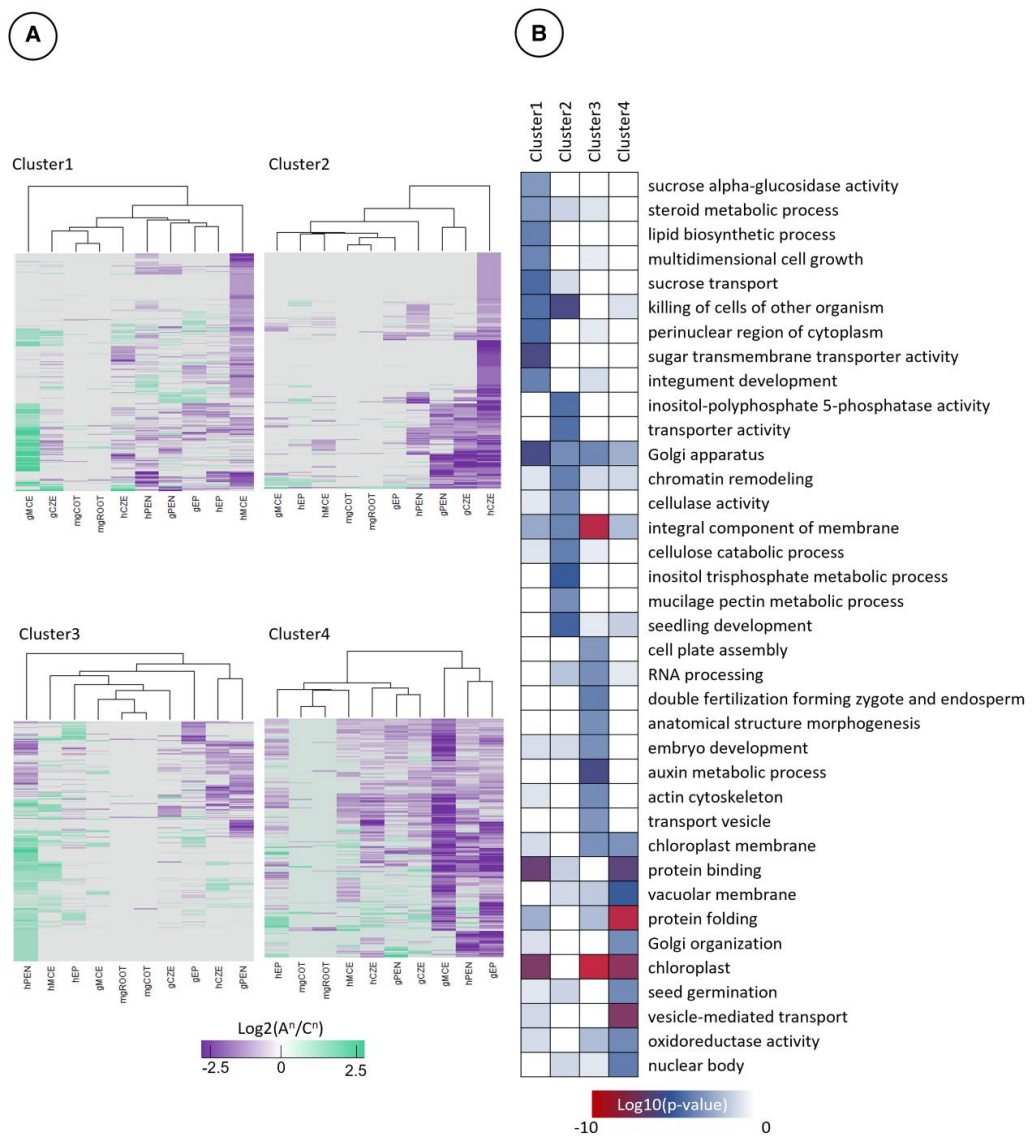
GO analysis of gene clusters in filial subregions. **A)** Clusters of the data in Fig. 8C, selected for exemplifying subgenome bias in certain stages or entire lack thereof. **B)** GO enrichment of the clusters selected in **A)**, wherein statistically significant GO terms were only selected if >1 gene homolog pair constituted the GO term. Complete GO analysis is documented within Supplementary Data Set S4.

Data clustering of gene homoeologs from the filial subregions revealed little subgenome bias at the whole transcriptome level (Fig. 8A; Supplementary Data Set S3). The mgCOT and mgROOT exhibited the least bias and were likely due in part to the low proportion of genes expressed in the mature embryo (Fig. 2A; Supplementary Fig. S4). The gEP clustered away from all other filial subregions, while the CZE clustered together throughout morphogenesis. The hEP and mgROOT/mgCOT also clustered together, implying the distribution of subgenome bias within the EP remains distinct from the endosperm subregions. Despite this, the hEP and mgROOT/mgCOT clustered adjacent to the hMCE and hPEN, suggesting slight similarity of subgenome bias in postglobular seeds. The CZE clustered together by subregion rather than by developmental stage, with both the gCZE and hCZE exhibiting similar bias in subgenome profiles. The gEP had the most gene pairs that had a high level of subgenome bias (Log_2_(A^n^/C^n^) ≥ 2 or Log_2_(A^n^/C^n^) ≤ −2), at 1,715 gene pairs (812 A^n^/903 C^n^). This comprised 11.3% of all identified homoeologous gene pairs with detectable expression of either gene, while only 8.2% of gene pairs were identified as “low bias” (0.1 ≥ Log_2_(A^n^/C^n^) ≥ −0.1). The mgCOT and mgROOT had the fewest genes with high bias (54 A^n^/47 C^n^ and 58 A^n^/45 C^n^, respectively), which collectively accounted for 2.3% and 2.8% of the homoeologs with detectable expression (Fig. 8B). All subregions aside from the mgCOT and mgROOT had more gene pairs with high C^n^ subgenome bias. For example, the gMCE, gPEN, and gCZE had 720, 497, and 502 gene pairs with high C^n^ bias, compared to 603, 375, and 441 biased toward the A^n^ subgenome, respectively. The data were then clustered into smaller groups for downstream analysis (Fig. 8, C and D). We focused on 4 clusters based on the same criteria described in Fig. 3, C and D. Clustering analysis revealed bias in the A^n^ subgenome of the gMCE and C^n^ bias of the hMCE in Cluster 1, whereas Cluster 2 formed a clade with distinct C^n^ subgenome bias of the syncytial endosperm subregions (gPEN, gCZE, and hCZE) (Fig. 9A). Cluster 3 identified A^n^ bias of the gPEN and a C^n^-biased clade of the hCZE and gPEN. Cluster 4 had low overall subgenome bias aside from the C^n^-biased clade that grouped the gMCE, hPEN, and gEP together. Cluster 1 was significantly enriched (*P* < 0.01) for GO terms associated with cell growth, polysaccharide transport and synthesis, Golgi apparatus, and steroid metabolism (Fig. 9B; Supplementary Data Set S4). Cluster 2 was also significantly enriched for biological processes implicated in Golgi apparatus function, as well as inositol metabolism, cellulose and cell wall processing, and chromatin modification. Cluster 3 was enriched in gene pairs associated with cell division, reproductive development, auxin metabolism, and actin cytoskeleton. Cluster 4 was significantly enriched in vacuolar membrane, vesicle transport, and protein folding GO terms. Notably, while data clustering of the filial regions identified distinct clades, the subregions at large did not exhibit similar genome-wide subgenome bias as seen in the maternal tissues. While the filial subregions have a greater proportion of gene pairs annotated as “strong bias” as compared to “low bias” pairs in our analysis, the maternal subregions had many more gene pairs with small but noticeable biases, as evident by comparing Figs. 3A and 8A. Altogether, this indicates the filial regions have a large proportion of transcripts that are only slightly biased to each subgenome. It is likely that subgenome bias plays a lesser role in global gene expression of the filial subregions than what is seen in maternal compartments.

### Endosperm cellularization is associated with C^n^ subgenome bias of genes involved in cell wall synthesis

**Figure 10.**
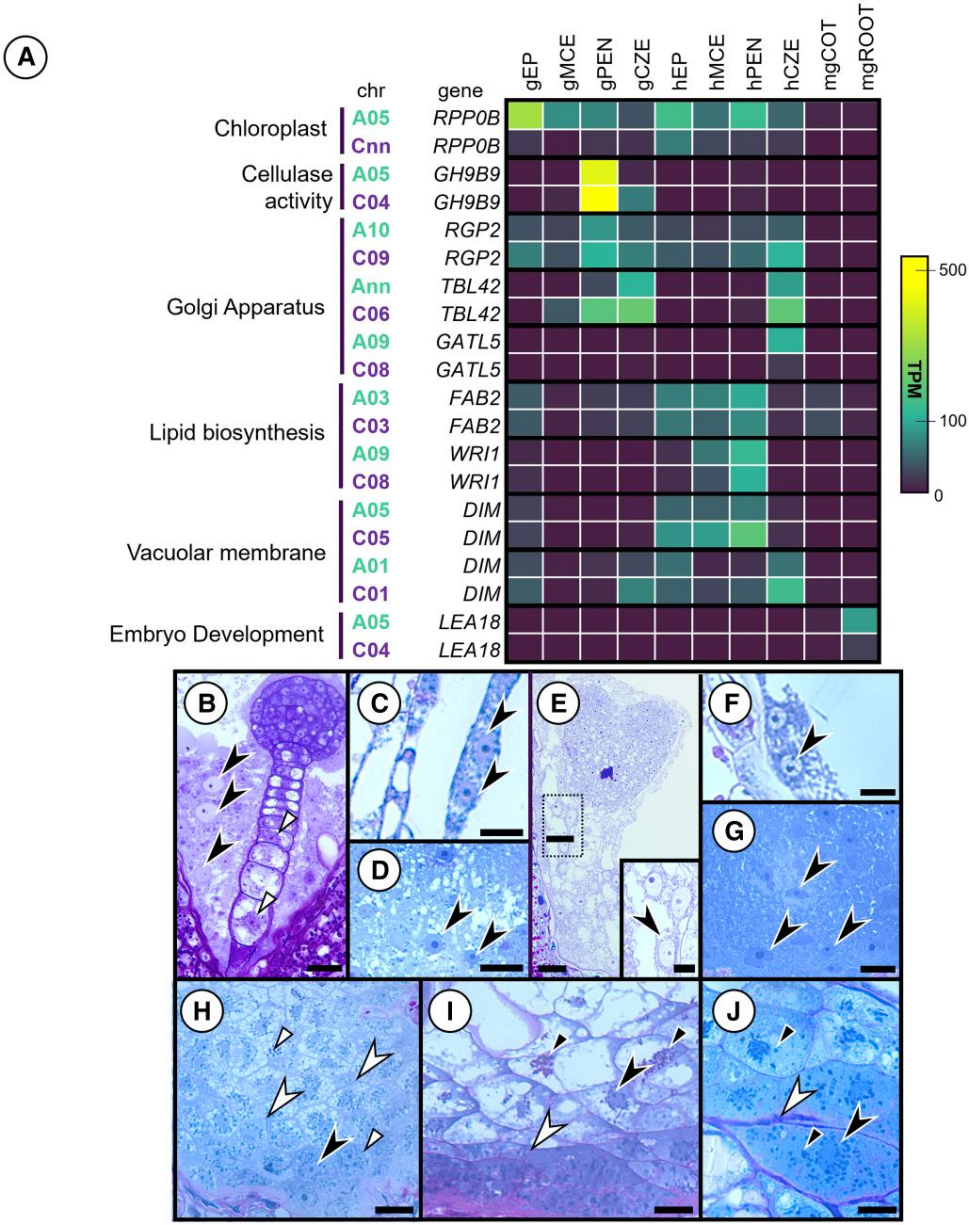
Transcription of genes associated with cell wall, endomembrane, and cellular modification in filial regions. **A)** Heat map of selected homoeologues belonging to significantly enriched GO terms from the clusters in Fig. 9. **B)** 7 DPP seed globular EP atop the suspensor at the micropylar pole, with enlarged nuclei (black arrowheads) in the MCE syncytium and abundant plastids (white arrows) particularly in the perinuclear region. **C)** The PEN at 7 DPP composed of free nuclei in the syncytium, continuous with the **D)** CZE. **E)** 10 DPP heart EP with enlarged nuclei in the MCE migrating closer together prior to cellularization. **F)** PEN 10 DPP with nuclei remaining in a syncytium, as in the **G)** CZE. **H)** CZE of D14 seeds initiate cell wall formation (white arrowheads), which isolate the nuclei of the syncytium. Perinuclear protein-rich plastids (white arrows) populate the CZE. **I)** CZE of D21 seeds establish complete cell walls, terminating the syncytium within the chalaza of the seed. Plastids distal to the CPT convert to starch storage (amyloplasts, black arrows). **J)** D28 seeds divide the CZE with robust cell primary wall thickenings. Bar = **B)** 40 *µ*m, **C)** 20 *µ*m, **D)** 40 *µ*m, **E)** 100 *µ*m, **E_inset_)** 50 *µ*m, **F)** 30 *µ*m, **G)** 30 *µ*m, **H)** 30 *µ*m, **I)** 30 *µ*m, and **J)** 20 *µ*m.

GO term enrichment identified that some genes implicated in cell wall formation and nutrient reservoir deposition were expressed only in some subregions and others only in 1 subgenome (Fig. 10A). For example, the C^n^ homoeolog of *REVERSIBLY GLYCOSYLATED POLYPEPTIDE 2* (*RGP2*) was more abundantly transcribed in the gPEN and hCZE. A similar pattern was observed in the expression of *TRICHOME BIREFRINGENCE-LIKE 42* (*TBL42*), a gene associated with pectin deposition in cell walls where the C^n^ homoeolog was highly transcribed in the gPEN, gCZE, and hCZE. In contrast, another gene involved in pectin biosynthesis, *GALACTURONOSYLTRANSFERASE 5* (*GATL5*), was transcribed largely from the C^n^ homoeolog in the A^n^ subgenome. Genes involved in lipid biosynthesis (*FAB2* and *WRI1*) were slightly more transcribed from the A^n^ subgenome. *DIM*, a gene involved in brassinosteroid biosynthesis, was observed to have bias in the C^n^ subgenome across both homoeologs in the hEP, hMCE, hPEN, and hCZE.

At the globular stage of development, the MCE was entirely syncytial, with free nuclei throughout the shared cytoplasm (Fig. 10B). At 7 DPP, the PEN and CZE also had discrete nuclei in the syncytium (Fig. 10, C and D). By 10 DPP, the nuclei enlarged and migrated closer together in preparation for cellularization (Fig. 10E), while the PEN and CZE retained the continuous syncytium (Fig. 10, F and G). By 14 DPP, cell plates were visible in the CZE with proteoplasts encircling the nuclei (Fig. 10H). Proteoplasts in the CZE converted to amyloplasts at 21 DPP (Fig. 10I), and by 28 DPP, thickened primary walls then surround the nuclei of the CZE, finalizing the cellularization of the CZE (Fig. 10J). Taken together, data analysis revealed subgenome bias to be minimal in the filial tissues compared to the maternal subregions. Thus, it is apparent that the wave of endosperm cellularization may be partially controlled by genes exhibiting subgenome bias, particularly genes involved in pectin deposition like *GATL5* or *TBL42* and cell wall construction like *GH9B9*. While our gene coexpression analysis did not identify previously known endosperm cellularization genes like *AGAMOUS-LIKE 62* (*AGL62*) or *ENDOSPERM DEFECTIVE 1* (*EDE1*) (Kang et al. 2008; Pignocchi et al. 2009), it does not preclude that these genes are affected by polyploidization.

### Putative paralogous genes involved in seed storage reservoirs exhibit divergent subgenome bias

**Figure 11.**
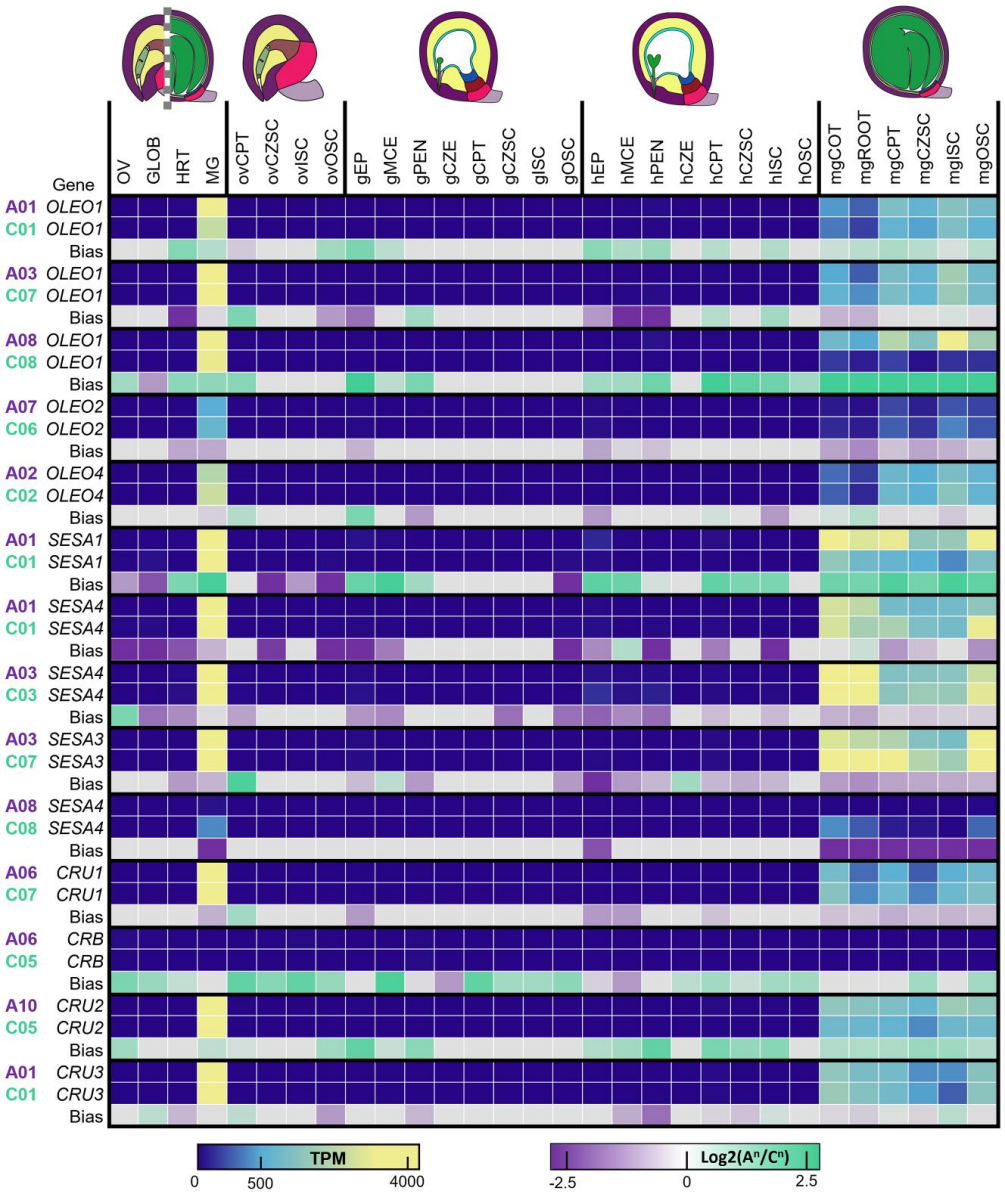
Transcript accumulation of *B. napus* homologs of known seed storage and seed oil biogenesis genes of *Arabidopsis*. Transcript accumulation represented in TPM and “bias” represented as (Log_2_(A^n^/C^n^) ≥ 2 (“A Bias”) or Log_2_(A^n^/C^n^) ≤ −2 (“C Bias”).

We investigated well-known seed storage protein family genes (*SEED STORAGE ALBUMIN* [*SESA*] and *CRUCIFERIN* [*CRU*]) and the OLEOSIN (*OLEO*) gene family to better understand if subgenome bias was prevalent across not only homoeologous pairs but also their putative paralogs. All *OLEO* genes are highly expressed late in seed development in both maternal and filial subregions and especially within the mgCOT, mgCPT, and mgISC (Fig. 11). All *OLEO* genes were more highly expressed in the mgCOT than in the mgROOT. Interestingly, a homoeologous gene pair of *OLEO1* was biased in A^n^ subgenome transcript activity across all subregions but the CZE in HEART seeds and in all subregions in MG seeds. The inverse trend was observed in *SESA4*, which was biased toward the C^n^ subgenome in 1 homoeologous pair than the other two. The C^n^-biased *SESA4* pair was the least expressed pair of *SESA* homoeologs. While most *CRU* genes were highly expressed in all seed subregions during maturation, *CRUCIFERIN B* (*CRB*) had comparably lower global expression while *CRUCIFERIN 2* (*CRU2*) was conspicuously A^n^ subgenome biased. As seeds are protected storage reservoirs for the developing embryo, the presence of subgenome bias or differences in transcription of putative gene paralogs pertaining to seed storage are notable for their influence on seed longevity and viability. *OLEO1*, *SESA4*, and *CRU2* all had putatively paralogous pairs that exhibited greater subgenome bias than observed in their partners. Thus, elements of seed storage in *B. napus* have been unevenly affected by polyploidization.

## Discussion

### Global transcriptome of the *B. napus* seed is biased toward the C^n^ subgenome in nearly all subregions

Dynamic changes in gene activity underpin development of the *B. napus* seed lifecycle. While C^n^ subgenome bias pervades throughout development, it is important to consider subgenome bias through many genome contexts including the number of genes expressed from each subgenome, which subgenome generates the most transcripts, and bias between subgenome gene homoeologs that contribute to the biases present during seed development. Our previous work showed C^n^ subgenome bias of DNA methylation and siRNA accumulation in the *B. napus* seed (Ziegler et al. 2023). Resynthesis of *B. napus* through hybridization of *B. rapa* and *B. oleracea* experiments report C^n^ subgenome bias in vegetative tissues is consistent across independently resynthesized individuals (Bird et al. 2021). It is also known that *B. napus* resynthesis is accompanied by substantial revision in parental gene expression (Wu et al. 2018). Here, we report that subgenomic bias is globally diminished as the seed matures, with the EP having notably fewer unique transcripts. Our previous work demonstrated that the epigenetic transition from morphogenesis to maturation was characterized by a global increase in DNA methylation in tandem with more siRNA loci being transcribed (Ziegler et al. 2023). These data, taken from the whole seed, implied that maturation was characterized by a global silencing effect on the whole genome. However, when specific compartments of the seed are dissected, and mRNAs profiled, the embryo has substantially fewer genes being transcribed with comparably similar transcript accumulation to that of other subregions of the seed. While recent research that profiled the transcriptomic bias of the embryo and seed coat reported similarly global C^n^ subgenome bias in *B. napus*, our work suggests that these biases are exacerbated when dissecting the maternal regions to cell and tissue-specific subregions (Gao et al. 2022). It is, however, important to note that *B. napus* has a complicated natural history leading to diverse genetic backgrounds. Resynthesized *B. napus* and the many domesticated cultivars will have different subgenomic balances. Emergent genome dominance should always be considered in the context of the cultivar and background. Furthermore, it has been proposed that the natural kinetics of transcription induce imbalanced gene expression in allopolyploids as the default condition (An et al. 2024), which reinforces genetic background as an important consideration in how gene expression bias manifests in individual genetic lines.

### The CPT of *Brassica* presents a unique opportunity to investigate the evolution of oilseeds

Seeds of the Brassicaceae have CPTs that undergo varying degrees of degeneration. For example, in *Arabidopsis* (Camelineae), the CPT is diminutive and ephemeral making it difficult to study. This is true of many clades within the Brassicaceae, including *Lepidium* (Lepidieae), *Capsella* (Camelineae), and *Thlaspi* (Thlaspideae) (Nguyen et al. 2000; Brown et al. 2004). *Brassica* (Brassiceae), *Erysimum* (Erysimeae), *Stanleya* (Thelypodieae), and *Descurainia* (Descurainieae) in contrast have a discrete CPT, which persists throughout late seed development. Few studies have interrogated the anatomy of the subregions in these groups, and details of gene activity underpinning its development are scant (Brown et al. 2004; Ziegler et al. 2019). In *B. napus*, the CPT is derived from the chalazal nucellus and undergoes dynamic changes in development as the seed matures. It has been established that the maternal–filial interface of the seed acts as a central integration hub of developmental signaling (Povilus and Gehring 2022). The CPT and CZE in *B. napus* are not segregated by a cuticle, unlike the junctions between the ISC and the PEN/MCE. Thus, cross-signaling is likely to occur through the chalazal pole, with the CPT acting as the conduit connecting the CZSC and CZE. The CPT then must adopt transcriptomic profiles to match the needs of the coordination between the maternal and filial subregions. Our results show that subgenome bias of homoeologous gene pairs of the ovCPT is dissimilar to other maternal subregions. The CPT is anatomically one of the most variable subregions of Brassicaceae seeds, and this variation is largely due to the persistence or degeneration of the nucellus, wherein persistent chalazal nucellar tissue differentiates to become distinct, or is entirely lost due to cellular elimination (Ingram and Penfield 2016). Given our data show that homoeologous gene pairs in the ovCPT are transcribed differently than they are in other subregions, it is possible polyploidization affects the nucellar development of crucifer seeds. As the robust CPT in *Brassica* likely imparts the ability to accrue nutrient reservoirs, this research could lead to important discoveries in oilseed evolution.

### Cell collapse of the ISC and CPT is an important requisite of seed maturation

Cell elimination and specifically those of the nucellus are crushed or undergo forced death to accommodate for nutrient transfer between the subregions of the seed (Lu et al. 2021). *Arabidopsis* seeds are comparably diminutive compared to other Brassicas yet still exclude portions of the nucellus through cellular elimination. This process opens the possibility of the seed coat to transfer sugars directly into the endosperm. The nucellar tissue is symplastically continuous with the seed coat in *Arabidopsis* and was previously documented in the chalaza of *Brassica* (Millar et al. 2015). Here, we demonstrate that cellular elimination permeates *B. napus* seeds and facilitates the formation of the pigment layer shortly after the CPT transitions from nucellar tissue. The chalazal maternal subregions (CZSC and CPT) must dictate the transition from somatic growth of the sporophytic tissue to megasporogenesis and finally female gametophyte formation. This occurs via the proliferation of the megasporangium inserted above the chalaza of the ovule, followed by the differentiation of the megaspore mother cell. This process relies on the *SPOROCYTLESS/NOZZLE* (*SPL*) signaling pathway, which has been documented in *Arabidopsis* to involve *WUSCHEL* (*WUS*), *TORNADO 2* (*TRN2*), *CIN-CINNATA* (*CIN*), and both *WIH1* and *WIH2* (Wei et al. 2015; Kaur et al. 2024). WIH1 and WIH2 have been shown to induce megasporogenesis after activation by WUS and are thus required for the differentiation of the MMC (Lieber et al. 2011). *WIH1* is highly expressed in the CZSC prefertilization and has high transcript accumulation in the C^n^ subgenome of the CPT and CZSC during seed morphogenesis. In early seed development, it seems likely that the CZSC and CPT control the formation of the female gametophyte and that the CPT of *Brassica* seeds is a subsection of cells derived from the nucellus after cellular elimination of the megasporangium. By maturation, the viable CPT cells are fully subsumed into the pigment layer and have undergone another transition. The method in which these cells are eliminated from both the nucellus (CPT) and the ISC is currently unknown. It is possible this degeneration is governed primarily by the mechanical expansion of the EP, but the synchronicity of the cellular elimination suggests coordinated cellular signals are likely involved. In the tapetum of anthers, *CEP1* is required for coordinated cellular degeneration immediately preceding anther dehiscence (Zhang et al. 2014). In *B. napus*, *CEP1* is largely expressed in the subregions that undergo coordinated cell elimination during seed development and is therefore possible that these processes could be conserved in carpellate reproductive tissues. Despite the CPT being ontogenetically related to the megasporangium, this dynamic subregion likely undergoes sequential modifications to suit the needs of ovule development and both seed morphogenesis and maturation.

### Seed coat subregions experience subgenome bias distinct from one another

The integuments serve to protect the developing nucellus by proliferating and elongating to encircle it. It is generally believed that the integuments arose as a form of either dichotomous branches or as lateral organs from the nucellus (Mathews and Kramer 2012; Meade et al. 2021). Despite their ancestral similarity, these structures have become anatomically and transcriptomically divergent with evolutionary time. Our data reveal that the ISC and OSC of *B. napus* exhibit different subgenomic biases across homoeologous genes, particularly during seed morphogenesis. Our anatomical data show that the ISC accumulates protein and oil bodies accompanied by thickened primary walls during maturation, whereas the OSC consumes much of its starch reservoirs, develops the palisade layer fully with reinforced pectin-rich walls, and deposits mucilage in the dermal cells. While much of these processes are known to occur in *Arabidopsis* (Beeckman et al. 2000; Tsai et al. 2017), our data indicate that these developmental transitions are accompanied by dissimilar subgenomic balance among the 2 seed coat layers during early seed development. The specialization of each seed coat layer is an important facet of seed development (Khan et al. 2014), and the prevalence of subgenome bias demonstrates that the biochemical programs required for these processes are subject to alterations due to polyploidization. In fact, resynthesis experiments of hexaploid *Brassica* have demonstrated that machinery relating to seed coat color undergo dramatic transcriptomic shifts postpolyploidization (Liu et al. 2020). Thus, given seed coat structure is critical for the protection of the embryo, resynthesis experiments could be used to improve seed coat quality or durability for food crops.

### Filial subregions experience less subgenomic bias than maternal subregions

*B. napus* subgenome bias is well documented to exist in many different tissues, including both siliques and whole seeds (Li et al. 2020; Khan et al. 2022; de Jong and Adams 2023). While we report conspicuous subgenomic bias characterizing the maternal subregions, the filial subregions are not subject to the same subgenomic asymmetry. At a global transcriptome level, large sets of gene pairs were of similar expression across subgenomes, particularly in the mgROOT and mgCOT. It is possible that the asymmetric silencing of 1 subgenome is alleviated upon establishment of the new sporophytic generation. The mgROOT and mgCOT both accumulate similar amounts of total transcripts as other subregions, yet fewer genes are transcribed altogether. This phenomenon may be due to mass erasure of gene activity in favor of a smaller subset of genes required for maintenance of the matured embryo. In particular, our data reveal that some of the most highly expressed genes in the mgROOT/COT encode SSPs and oleosin family proteins at several orders of magnitude higher expression than the vast majority of all other genes. This dramatic reduction in transcript activity is not reflected in mature *Arabidopsis* embryos (Belmonte et al. 2013). It is likely that the majority of all gene activity in the *B. napus* mgEP is dedicated to seed storage where the influence of polyploidy in the mature embryo is unlikely to be influential outside of seed storage.

### Seed storage reservoirs have divergent expression patterns within homoeologous gene pairs

The process of seed maturation is essential to the longevity of the seed, and many genes have been identified which enable storage of energy-dense proteins or lipids (Nguyen et al. 2015; Niñoles et al. 2022). Oleosins are required for the maintenance of lipid droplets, and seed storage albumins and cruciferins are known to be essential seed storage proteins (Miquel et al. 2014; Zheng et al. 2022). Despite the high transcript abundance in mature embryos, these genes experience exponential differences in expression across subgenomes. For example, 1 homoeolog pair of *OLEO1* is expressed a minimum of 6.5× more in the A^n^ subgenome in the mgROOT and a maximum of ∼39× more highly transcribed from the A^n^ subgenome in the mgCZSC. Furthermore, these differences occur within homoeologous gene pairs, including *OLEO1*, *SESA1*, *SESA4*, and *CRU2*. Despite the numerous copies *B. napus* has of these genes, only certain homoeologous pairs experience this substantial bias between the 2 subgenomes. This implies that seed storage genes experience subgenome dominance in certain homoeologs, but this bias does not occur evenly across all putatively paralogous genes.

## Conclusions

Our work provides evidence of subgenome bias across seed development at the cell and tissue levels of the newly formed allotetraploid *B. napus*. Maternal subregions like the CPT and CZSC elicit a more varied palette of subgenome bias than the comparatively stable subregions of filial seed. The previously unexplored maternal CPT is of particular interest to crop development in oilseeds and is potentially a critical aspect of Brassicacaeae seed diversity. Cells and tissues of the seed that are of maternal origin would benefit from additional studies through the lens of polyploidy, both within established oilseeds in addition to their close relatives or progenitors. Further interrogation of individual compartments over time will provide additional insight into the genetic circuits underpinning reproductive development at the cell and tissue-specific levels. Together, we find that individual cells, tissues, and organs across the seed lifecycle are influenced by polyploidization in ways distinct from each other and cooperate in the making of a seed.

## Materials and methods

### Plant growth conditions and collection

*B. napus* cv. Topas plants were grown in growth chambers under long-day conditions (16 h light, 8 h dark, 22 °C, relative humidity 50% to 70%). Seeds were germinated in soil, and plants were grown in a peat-based medium (1-gallon pots, Sunshine Mix #1). Flowers that had opened the same day were tagged and hand pollinated between 15:00 and 17:00,—8 to 11 h after the photoperiod began (Chan and Belmonte 2013). Seeds were collected at 0 (OV), 7 (GLOB), 10 (HEART), and 28 (MG) DAP for LMD and 0, 7, 10, 14, 21, and 28 DAP for histological analysis. Flower pollination was performed shortly after anthesis, when the anthers of the flower barely exceeding the corolla. Flower and silique harvest were performed between 15:00 and 17:00 to minimize time of day effects on gene activity.

### LMD

We used LMD to capture individual subregions of the seed. All tissue processing and collection were performed under RNase-free conditions according to the methods of Ziegler et al. (2019). Siliques of OV, GLOB, HEART, and MG stage seeds were cut at 50-mm fragments and fixed in 1:3 (*v*/*v*) glacial acetic acid:85% ethanol for 24 h at 4 °C. Tissues were then dehydrated in a graded ethanol series, followed by a transition infiltration with xylenes at 4 °C and gradually infiltrated with paraffin at 60 °C. Samples were incubated for <8 h at 60 °C prior to embedding in 100% paraffin wax (McCormick Scientific, Paraplast Plus). Serial sections (7-*μ*m sections, Leica RM2245 rotary microtome) were mounted on polyethylene napthalate (PEN) membrane slides (Leica Microsystems). Slides were deparaffinized in xylenes for 1 min, and seed subregions were isolated using the Leica Laser Microdissection 7000 system.

### RNA isolation

Total RNA was extracted from the laser-microdissected subregions using the Ambion RNaqueous micro kit and treated with Qiagen RNAase-free DNase to remove genomic DNA. Each replicate was a pool of a minimum of 100 histological sections from at least 12 different plants. We sequenced between 2 and 5 replicates of each subregion (ISC, OSC, CZSC, CPT, MCE, PEN, CZE, and EP). A detailed replicate list is available in Supplementary Data Set S5. This method of pooling has been shown to be sufficient in previous spatial transcriptomic work (Belmonte et al. 2013; Cervantes-Pérez et al. 2024). RNA quantity was evaluated using the Quant-iT RiboGreen RNA Assay kit (Invitrogen), and quality was evaluated using the Agilent 6000 Pico LabChip and the Agilent 2100 bioanalyzer (Agilent Technologies, United States). A minimum RIN of 3 was required for further cDNA synthesis and library preparation (Belmonte et al. 2013).

### Library preparation and sequencing

First and second cDNA synthesis was performed according to a high-throughput RNA-seq protocol (Kumar et al. 2012). Library preparation was performed using the NuGen Ovation Ultralow DR Multiplex System. To validate sample quantity, we used the Quant-iT PicoGreen dsDNA Assay Kit (Invitrogen), qPCR, and the High Sensitivity DNA Analysis Kit (Agilent) on the Agilent 2100 Bioanalyzer according to the manufacturer's instructions. Libraries were pooled and size selected (350 to 600 bp) on the E-Gel system (Invitrogen). mRNA-seq libraries were then sequenced on the Illumina HiSeq 2500 platform at Genome Québec. Whole seed RNA was extracted from whole globular stage seeds using PureLink Plant RNA Reagent (Ambion).

### RNA-seq analysis

Low-quality reads and adaptors were removed from the RNA-seq data set with fastp (Chen et al. 2018). Surviving reads were then aligned to the *B. napus* cv. ‘Darmor-bzh’ genome assembly from Chalhoub et al. (2014) with HISAT2 (Kim et al. 2019). WGS contigs from Chalhoub et al. (2014) at accessions CCCW010000001 through CCCW010044187, and scaffolds, annotation data at accessions LK031787 to LK052685 in the European Nucleotide Archive were used for read alignments and RNA-seq analysis. Quantification of uniquely mapped reads was performed using featureCounts, native to the subread package (Liao et al. 2019). Counts were then normalized to TPM. TPM data table can be found in Supplementary Data Set S6. Data clustering and heat map visuals were completed using heat map(r), and Euclidean distance was used to compute distance between nodes during clustering. Gene homoeologs were identified as annotated within Gao et al. (2022). We used a quotient of Log_2_(A^n^/C^n^) as a metric to quantify relative bias in gene homoeolog expression. This ratio was used to visualize differences in gene expression between predicted homoeologs. We observed that most gene homoeologs that exhibited substantial differences in expression between the 2 subgenomes were often ≥4-fold TPM greater in 1 subgenome than the other. Consequently, we used Log_2_(A^n^/C^n^) ≥ 2 or Log_2_(A^n^/C^n^) ≤ 2 as our cutoff to cluster the gene lists. Clustered data were transformed into data matrices using matTree, and GO enrichment was performed on the gene lists using SeqEnrich (Becker et al. 2017) to determine statistical significance of potential biological processes influenced by polyploidy. Additionally, we created a Canola eFP Browser for visual comparisons of gene expression across seed subregions, and this can be accessed at https://bar.utoronto.ca/efp_canola/cgi-bin/efpWeb.cgi. Transcript data on a per homoeolog basis were summarized/standardized using the TPM method and were databased on the Bio-Analytic Resource for Plant Biology server in an MySQL database. Raw sequencing data are available on Gene Expression Omnibus (GEO) as GSE271809 and through the Sequence Read Archive (SRA) (PRJNA1065514). Replicates of each subregion and developmental stage are matched to SRA accession in Supplementary Data Set S5.

### Light microscopy analysis

Samples were fixed in 1.6% glutaraldehyde and 4% paraformaldehyde solution overnight and then transferred to methylcellosolve for 6 to 18 h (older seeds require longer time and more thorough dehydration). Samples were then transferred into ethanol 3 consecutive times, with each wash lasting 24 h. Tissue was then infiltrated with activated Leica HistoResin over 3 d (1/3 ethanol to HistoResin, followed by 2/3 and then 100% HistoResin). Samples were then embedded onto pucks and sectioned at 2.5-*µ*m thickness using a Leica RM2245 rotary microtome with a steel blade and mounted on glass slides. All slides were stained with periodic acid-Schiff's reagent and then followed with either toluidine blue O or amido black. Slides were sealed with Cytoseal 60 (Richard-Allan Scientific). Leica DM2500 light microscope equipped with a Leica DFC425 camera using LAS3.7 software was used to visualize the sections.

### Hand sectioning

Lipid staining was performed on live tissue sectioned by hand using double-edge razors. GLOB, HEART, and MG seeds were sectioned longitudinally and stained with Sudan IV for at least 25 min. Sections were mounted in glycerol to visualize.

### Transmission electron microscopy

Transmission electron microscopy was performed as described within Chan and Belmonte (2013) and Millar et al. (2015). Ovules and seeds were fixed overnight in 3% glutaraldehyde in 0.025 m cacodylate buffer supplemented with 5 mm calcium chloride (pH 7.0). Plant material was rinsed with cacodylate buffer and postfixed with 2% osmium tetroxide in 0.8% KFe(CN)_6_ in cacodylate buffer. After postfixation, seeds were rinsed with distilled water and stained overnight with a 0.5% aqueous uranyl acetate solution to increase contrast.

Processed seeds were rinsed in distilled water and dehydrated in a graded ethanol series. Seeds were then further dehydrated in 1:1 absolute ethanol to propylene oxide (*v*:*v*) and then in 100% propylene oxide. Finally, they were gradually infiltrated and embedded in Spurr's epoxy resin at 70 °C. All methods prior to embedding were performed at 4 °C. Using a Reichert–Jung Ultracut ultramicrotome, sections were cut (9- nm thickness) with a Diatome diamond knife and mounted on copper grids. The sections were visualized with a Hitachi H-7000 transmission electron microscope at 75 kV, and pictures were taken using AMT Image Capture Engine version 601.384.

### Accession numbers

Replicate data and SRA accession of each library can be found in Supplementary Data Set S5. *B. napus* cv. Darmor-bzh identifiers for genes in Figs. 5A, 6A, 7A, 10A, and 11 are found in Supplementary Data Set S7.

## Supplementary Material

kiaf283_Supplementary_Data

## Data Availability

Sequence data from this article can be found in the GEO data libraries as GSE271809 and within the SRA (PRJNA1065514).

## Supplementary data

The following materials are available in the online version of this article.

**Supplementary Figure S1.** Principal component analysis comparing *B. napus* seed subregions.

**Supplementary Figure S2.** Example subregion-enriched genes demonstrating LMD specificity and minimal contamination between subregions.

**Supplementary Figure S3.** Initial data clustering of maternal seed subregions.

**Supplementary Figure S4.** Initial data clustering of filial seed subregions.

**Supplementary Data Set S1.** Gene pairs from data clustering of maternal seed subregions*.

**Supplementary Data Set S2.** GO enrichment output of gene clusters from maternal subregions specified in Fig. 4A*.

**Supplementary Data Set S3.** Gene pairs from data clustering of maternal seed subregions*.

**Supplementary Data Set S4.** GO enrichment output of gene clusters from filial subregions specified in Fig. 9A.

**Supplementary Data Set S5.** Replicates used in the work matched to SRA accession.

**Supplementary Data Set S6.** TPM table of averaged expression values of all seed subregions across all developmental stages.

**Supplementary Data Set S7.**  *B. napus* cv. Darmor-bzh identifiers of genes in Figs. 5A, 6A, 7A, 10A, and 11.

**Supplementary File S1.** README supplement: Gene Expression landscape of the *B. napus* seed reveals subgenome bias in both space and time.

**See*  **Supplementary File S1**  *(README supplement) for more information detailing these data sets*.

## Dive Curated Terms

The following phenotypic, genotypic, and functional terms are of significance to the work described in this paper:

CRU2 Gramene: AT1G03880
CRU2 Araport: AT1G03880

GATL5 Gramene: AT1G02720
GATL5 Araport: AT1G02720

OLEO4 Gramene: AT3G27660
OLEO4 Araport: AT3G27660

UGE1 Gramene: AT1G12780
UGE1 Araport: AT1G12780

agl62 Gramene: AT5G60440
agl62 Araport: AT5G60440

ATS3 Gramene: AT5G07190
ATS3 Araport: AT5G07190

CRb Gramene: AT1G09340
CRb Araport: AT1G09340

EDE1 Gramene: AT2G44190
EDE1 Araport: AT2G44190

EXL3 Gramene: AT5G51550
EXL3 Araport: AT5G51550

MIRO3 Gramene: AT3G05310
MIRO3 Araport: AT3G05310

OLEO1 Gramene: AT4G25140
OLEO1 Araport: AT4G25140

XTH9 Gramene: AT4G03210
XTH9 Araport: AT4G03210

WRI1 Gramene: AT3G54320
WRI1 Araport: AT3G54320

trichome Gramene: NCBI
trichome Araport: NCBI

SWEET1 Gramene: At1g21460
SWEET1 Araport: At1g21460

SWEET10 Gramene: At5g50790
SWEET10 Araport: At5g50790
