# Supplementary material for: Gene expression landscape of the Brassica napus seed reveals subgenome bias in both space and time
Source: Plant Physiol. 2025 Jun 28;198(3):kiaf283. doi: 10.1093/plphys/kiaf283 (PMC12305542; doi:10.1093/plphys/kiaf283)
Supplement: kiaf283_Supplementary_Data [file kiaf283_supplementary_data.zip › PLPHYS-2025-0342R1__Supplementary Figures.pdf]

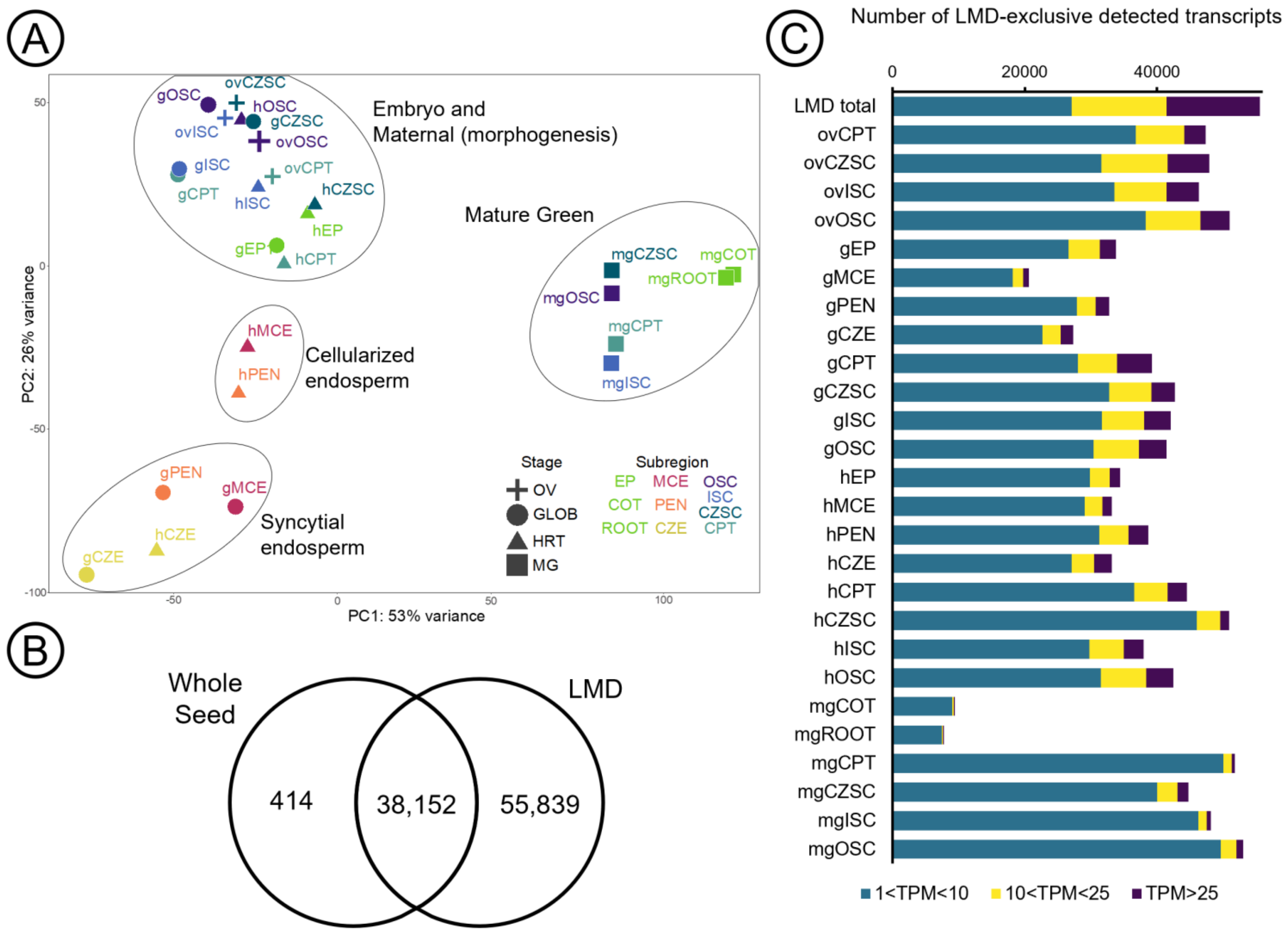

Supplementary Figure S1. (A) Principal Component Analysis (PCA) comparing the transcriptome profiles of the *Brassica napus* seed subregions. Each stage is marked with a unique identifier (OV = plus sign, GLOB = circle, HRT = triangle, MG = square). Each subregion is colour-coded as in Figure 1. (B) Transcripts detected (TPM>1) in whole seed and laser-microdissected seed transcriptomes over the OV, GLOB, HRT, and MG stages. Increasing spatial resolution yields 44% greater transcript detection in the developing B. napus seed. (C) Transcripts detected only by LMD-RNAseq in the developing B. napus seed. Transcripts are sorted into low (1<TPM≤10, teal), medium (10<TPM≤25, yellow), and high (TPM>25,) accumulation levels.

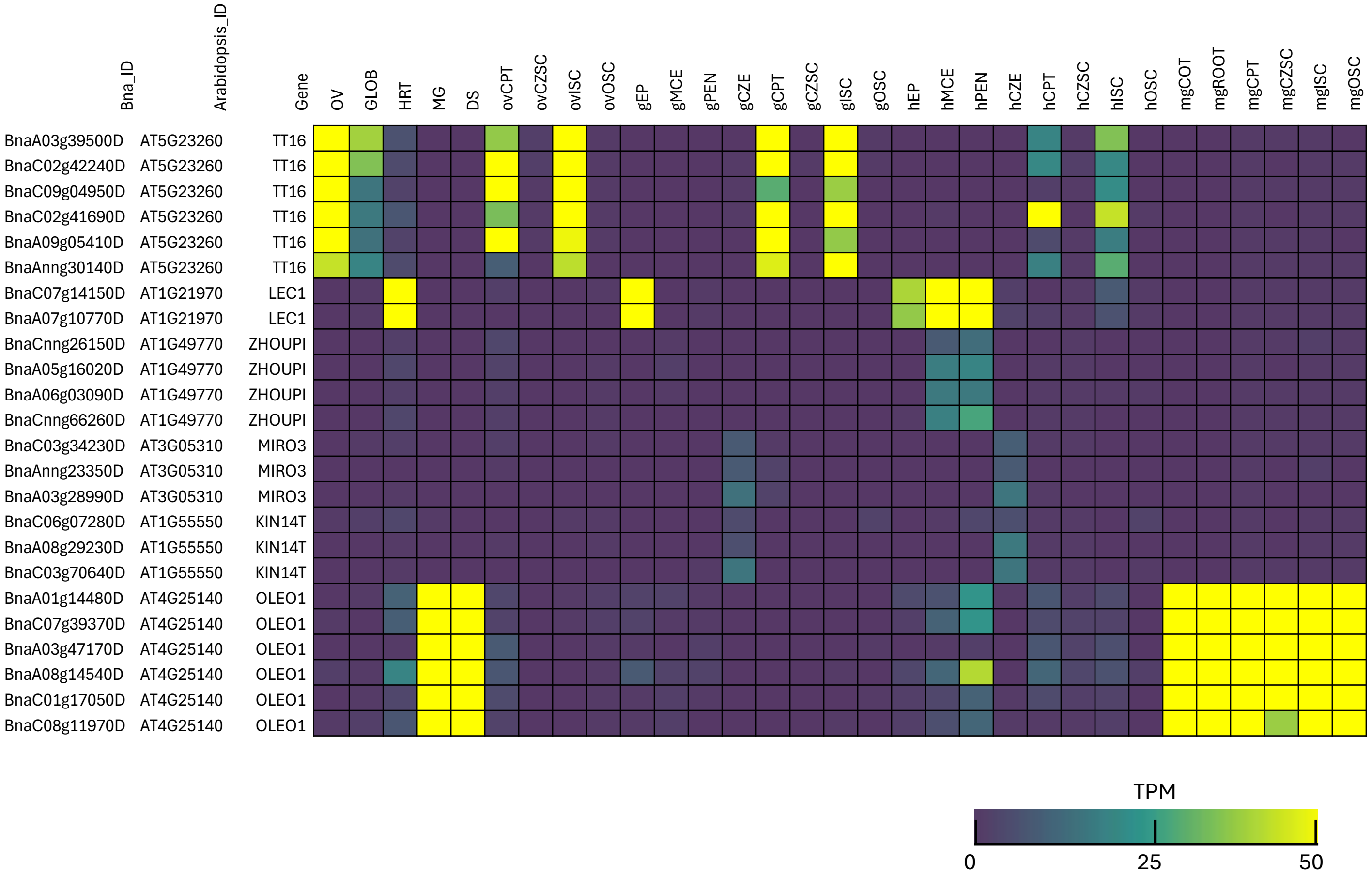

Supplementary Figure S2. Example subregion-enriched genes demonstrating LMD specificity and minimal contamination between subregions. Heat map colour is scaled based on highest and lowest values of each set of gene homologs.

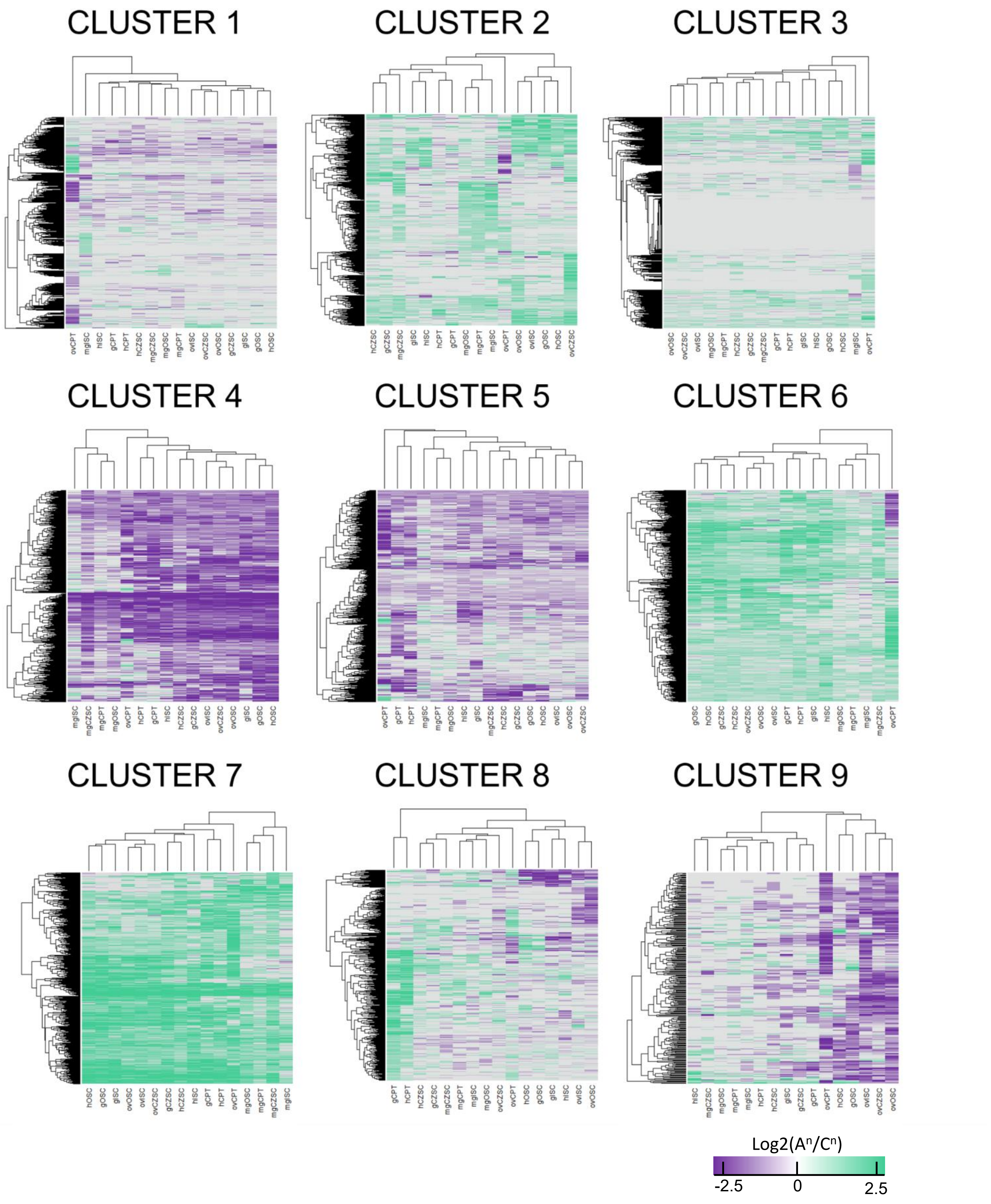

Supplementary Figure S3. Initial data clustering of maternal subregions, computed using Euclidean distance at k=4 using R cutree. Clusters 1, 2, 3, 6, and 8 were divided into smaller clusters to k=5, k=6, k=5, k=4, and k=3 clusters. Full breakdown of the gene lists within these clusters are within Dataset S1.

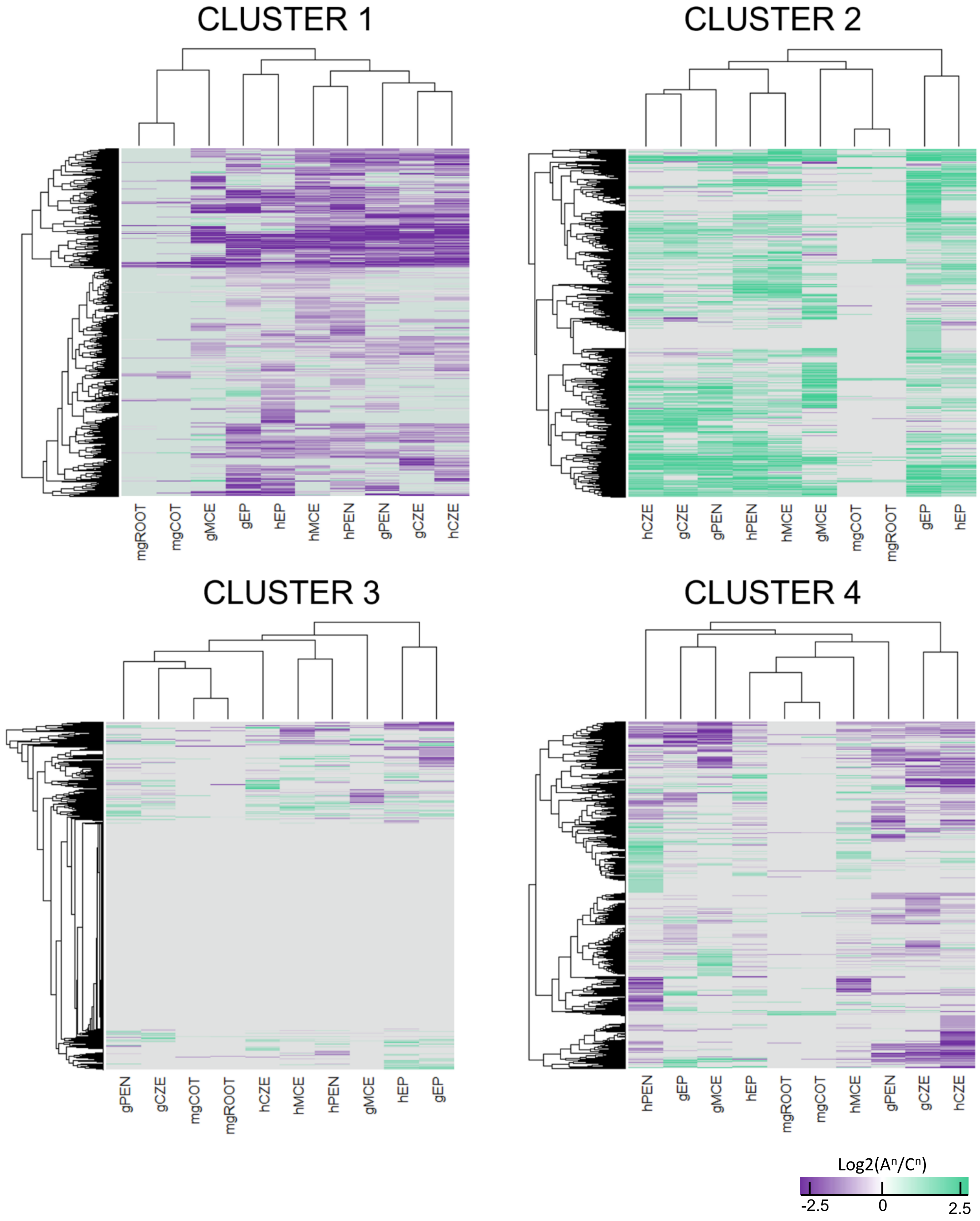

Supplementary Figure S4. Initial data clustering of filial subregions, computed using Euclidean distance at k=3 using R cutree. Clusters 1, 2, 3, 6, and 8 were divided into smaller clusters to k=2, k=3, and k=7 clusters. Full breakdown of the gene lists within these clusters are within Dataset S2.
